# Network reconstruction for trans acting genetic loci using multi-omics data and prior information

**DOI:** 10.1186/s13073-022-01124-9

**Published:** 2022-11-07

**Authors:** Johann S. Hawe, Ashis Saha, Melanie Waldenberger, Sonja Kunze, Simone Wahl, Martina Müller-Nurasyid, Holger Prokisch, Harald Grallert, Christian Herder, Annette Peters, Konstantin Strauch, Fabian J. Theis, Christian Gieger, John Chambers, Alexis Battle, Matthias Heinig

**Affiliations:** 1 Institute of Computational Biology, German Research Center for Environmental Health, HelmholtzZentrum München, Neuherberg, Germany; 2 German Heart Centre Munich, Department of Cardiology, Technical University Munich, Munich, Germany; 3 Department of Informatics, Technical University of Munich, Garching, Germany; 4 Department of Computer Science, Johns Hopkins University, Baltimore, MD USA; 5 Research Unit of Molecular Epidemiology, German Research Center for Environmental Health, HelmholtzZentrum München, Neuherberg, Germany; 6 Institute of Genetic Epidemiology, German Research Center for Environmental Health, HelmholtzZentrum München, Neuherberg, Germany; 7 IBE, Faculty of Medicine, LMU Munich, 81377 Munich, Germany; 8 Institute of Medical Biostatistics, Epidemiology and Informatics (IMBEI), University Medical Center, Johannes Gutenberg University, Mainz, Germany; 9 Department of Internal Medicine I (Cardiology), Hospital of the Ludwig-Maximilians-University (LMU) Munich, Munich, Germany; 10 Institute of Human Genetics, School of Medicine, Technische Universität München, Munich, Germany; 11 Institute of Epidemiology, German Research Center for Environmental Health, HelmholtzZentrum München, Neuherberg, Germany; 12 German Center for Diabetes Research (DZD), Neuherberg, Germany; 13 Institute for Clinical Diabetology, German Diabetes Center, Leibniz Center for Diabetes Research at Heinrich Heine University, Düsseldorf, Germany; 14 Division of Endocrinology and Diabetology, Medical Faculty, Heinrich Heine University, Düsseldorf, Germany; 15 Chair of Genetic Epidemiology, IBE, Faculty of Medicine, LMU Munich, Munich, Germany; 16 Department of Mathematics, Technical University of Munich, Garching, Germany; 17 Department of Epidemiology and Biostatistics, MRC-PHE Centre for Environment and Health, School of Public Health, Imperial College London, London, UK; 18 Lee Kong Chian School of Medicine, Nanyang Technological University, 308232 Singapore, Singapore; 19 Department of Biomedical Engineering, Johns Hopkins University, Baltimore, MD USA; 20 Munich Heart Association, Partner Site Munich, DZHK (German Centre for Cardiovascular Research), 10785 Berlin, Germany

**Keywords:** Systems biology, Multi-omics, Data integration, Network inference, Prior information, Simulation, Machine learning, Personalized medicine

## Abstract

**Background:**

Molecular measurements of the genome, the transcriptome, and the epigenome, often termed multi-omics data, provide an in-depth view on biological systems and their integration is crucial for gaining insights in complex regulatory processes. These data can be used to explain disease related genetic variants by linking them to intermediate molecular traits (quantitative trait loci, QTL). Molecular networks regulating cellular processes leave footprints in QTL results as so-called *trans*-QTL hotspots. Reconstructing these networks is a complex endeavor and use of biological prior information can improve network inference. However, previous efforts were limited in the types of priors used or have only been applied to model systems. In this study, we reconstruct the regulatory networks underlying *trans*-QTL hotspots using human cohort data and data-driven prior information.

**Methods:**

We devised a new strategy to integrate QTL with human population scale multi-omics data. State-of-the art network inference methods including *BDgraph* and *glasso* were applied to these data. Comprehensive prior information to guide network inference was manually curated from large-scale biological databases. The inference approach was extensively benchmarked using simulated data and cross-cohort replication analyses. Best performing methods were subsequently applied to real-world human cohort data.

**Results:**

Our benchmarks showed that prior-based strategies outperform methods without prior information in simulated data and show better replication across datasets. Application of our approach to human cohort data highlighted two novel regulatory networks related to schizophrenia and lean body mass for which we generated novel functional hypotheses.

**Conclusions:**

We demonstrate that existing biological knowledge can improve the integrative analysis of networks underlying *trans* associations and generate novel hypotheses about regulatory mechanisms.

**Supplementary Information:**

The online version contains supplementary material available at 10.1186/s13073-022-01124-9.

## Background

Genome-wide associations studies (GWAS) have been tremendously successful in discovering disease associated genetic loci. However, establishing causality or obtaining functional explanations for GWAS SNPs is still challenging. In recent years, the focus has shifted from discovery of disease loci to mechanism and explanation and large efforts have been put into unraveling the functional consequences of GWAS SNPs [1–4]. Technological advances in measuring molecular data led to a steady increase in biological resources providing simultaneous measurements of different types of molecules from the same individual. These include readouts of the genome (genotypes), the transcriptome (RNA abundance), and the epigenome (e.g., DNA methylation levels), yielding data commonly referred to as *multi-omics* data.

To elucidate disease mechanisms, systems genetics approaches link GWAS SNPs to intermediate molecular traits by identifying quantitative trait loci (QTL) [5, 6], for example for gene expression levels (eQTL) [7–9] or DNA methylation at CpG dinucleotides (meQTL) [10–12].

Genetic variants that are QTL for quantitative molecular phenotypes that reside on a different chromosome are called *trans*-QTL. Previously, *trans*-QTL studies were successful in model systems [13, 14]. More recently, large-scale meta analyses of molecular QTL in very large sample sizes have been applied to successfully map large numbers of *trans*-QTL in humans [9, 12]. These are particularly interesting as they have been found to be enriched for disease associations [9, 10, 15]. Yet, the underlying mechanisms leading to such associations can usually not be explained in a straightforward way [8]. In fact, in a recent study most discovered blood *trans*-eQTL in human could not be explained [9].

*Trans*-QTL *hotspots* are genetic loci which influence numerous methylation or expression levels of genes on different chromosomes [16]. Such coordinated effects can for instance be orchestrated through *trans* regulator genes encoded at the hotspot and further be propagated through regulatory networks involving protein-protein and/or transcription factor bindings. *Trans*-QTL hotspots can therefore be seen as footprints of regulatory molecular networks in the results of association studies and likely encode genomic master regulators. One way of mechanistically explaining the effects of these master regulators is by reverse engineering the regulatory networks and hence determining the intermediate molecular processes giving rise to the observed *trans* effects. This ultimately yields novel insights into disease pathophysiology [1, 16–18].

A large body of work has focused on inferring regulatory interactions from high-throughput data by individually combining different omics data like gene expression levels and genotype [8, 19–23] or chromosomal aberration [24] information. Generally, network inference to uncover regulatory mechanisms in biological systems has gotten much interest [17, 25–28]. The emergence of multi-omics data now also allows for establishing networks across more than two omics layers in a holistic approach to obtain more insight into the function of regulatory elements [18]. For instance, Bayesian networks have been applied to a collection of different data types in yeast to successfully reconstruct regulatory networks [29]. Major efforts have been made to recover functional interactions from such data, but methods to successfully reverse engineer regulatory networks across multiple omics layers are still lacking [1, 6, 30, 31].

Furthermore, utilizing the wealth of data available from genomic databases as biological prior information can guide the inference of complex multi-omics networks [31–33]. For instance, using known relationships discovered in previous studies as prior knowledge, such as protein-protein interactions (PPIs) or eQTL, can facilitate network reconstruction on novel datasets. This information can be utilized as edge-specific “weights” or “penalties” during the inference process by methods such as *BDgraph* [34] or the *graphical lasso* [35], respectively (more details below).

Application of priors has been investigated in numerous works (e.g., [17, 32, 34, 36–40]). While several studies show the advantage of using priors in synthetic datasets [26, 37, 39, 40] or model systems [17, 38, 40, 41], relatively few studies apply their inference methodologies to functional genomics data in humans [34, 39, 42, 43]. In case human data are considered, either cell line data are used [42], the inference is restricted to a single pathway [43], or no informative priors are used for this specific context [34]. Zuo et al. apply prior based inference to human cancer gene expression data; however, they only use priors based on PPIs extracted from the STRING database and focus on differential expression analysis [39]. What is still missing is to comprehensively integrate the vast amount of functional data from large-scale databases [44–47] as prior information in human multi-omic *trans*-QTL studies and to determine the appropriate inference methods.

Here, we developed a novel approach for understanding the molecular mechanisms underlying the statistical associations of *trans*-QTL hotspots by integrating existing biological knowledge and multi-omics cohort data to infer regulatory networks. We derived a comprehensive set of continuous priors from public datasets such as GTEx, the BioGrid, and Roadmap Epigenomics and applied state-of-the-art network inference methods including graphical lasso [35], BDgraph [34], GeneNet [48, 49], GENIE3 [50], and iRafnet [38]. These priors and methods were then applied to (1) simulated and (2) real world cohort data from the KORA and LOLIPOP cohorts, encompassing genotype, gene expression, and DNA methylation data. We further provide a proof-of-concept application of our approach to genotype and gene expression data originating from skeletal muscle tissue to showcase that it also translates to other contexts.

## Methods

### Cohort descriptions

In our study, we used cohort data from the KORA [51–53] and LOLIPOP studies. Both are population based studies with no selection for particular phenotypes at enrollment. More details can be found in the individual sections below and in the referenced original publications.

#### Cooperative Health Research in the Region of Augsburg (KORA)

KORA (Cooperative Health Research in the Region of Augsburg) is a research platform of independent population-based health surveys and subsequent follow-up examinations of individuals of German nationality resident in the region of Augsburg in southern Germany [51–53]. Written informed consent was obtained from all participants and the studies have been approved by the ethics committee of the Bavarian Medical Association. The present study is based on a subsample of 683 participants of the KORA F4 survey with methylation, expression, and genotyping data available (347 males and 336 females aged 62 to 81 years, median age 69) [54]. Study design, sampling method, and data collection have been described in detail elsewhere [51–53].

#### The London Life Sciences Prospective Population Study (LOLIPOP)

LOLIPOP is a prospective cohort study of  28K Indian Asian and European men and women recruited from the lists of 58 General Practitioners in West London, UK, between 2003 and 2008 [55]. The LOLIPOP study is approved by the National Research Ethics Service (07/H0712/150), and all participants gave written informed consent. At enrollment, all participants completed a structured assessment of cardiovascular and metabolic health, including anthropometry, and collection of blood samples for measurement of fasting glucose, insulin and lipid profile, HbA1c, and complete blood count with differential white cell count. Participants have been followed for incident health events, and 13,347 have attended clinical follow-up visits during which further blood samples were collected. The present study is based on a subsample of 612 participants of the LOLIPOP study with methylation, expression, and genotyping data available (259 males and 353 females aged 27.67 to 74.92 years, median age 55.17) [55].

### Cohort data processing

Methylation data were measured using the Infinium Human Methylation 450K BeadChip in both the KORA and the LOLIPOP cohort and methylation beta values obtained as described previously [54, 56]. Quantile normalized methylation beta values were adjusted for Houseman blood cell-type proportion estimates and the first 20 principal components calculated on the array control probes by using residuals of the following linear model:

$$methylation~\beta \sim 1 + CD4T + CD8T + NK + BCell + Mono + PC1 + \dots + PC20$$

For expression data, the Illumina HumanHT-12 v3 and Illumina HumanHT-12 v4 expression BeadChips were used in KORA and LOLIPOP, respectively, and processed as described previously [12, 57]. Only probes common to both arrays were selected for analysis. Expression data were adjusted for potential confounders by regressing log2 transformed expression values against age, sex, and RNA integrity number (RIN) as well as RNA amplification plate (KORA)/RNA conversion batch (LOLIPOP) (batch1) and sample storage time (KORA)/RNA extraction batch (LOLIPOP) (batch2) and obtaining the residuals from the linear model:

$$\begin{aligned} expression \sim age + sex + RIN + batch1 + batch2 \end{aligned}$$

Additional details on the cohort data and design are presented in [51–54, 57] (KORA) and [55, 56, 58] (LOLIPOP).

For the inference of the GTEx skeletal muscle-related network, we used GTEx v8 skeletal muscle data [59]. Potential confounders including first 5 genotype PCs, 60 expression PEER factors and measured covariates “WGS sequencing platform” (HiSeq 2000 or HiSeq X), “WGS library construction protocol” (PCR-based or PCR-free), and donor sex were removed from expression data prior to analysis. Processing has been performed as previously described and details can be found elsewhere [59].

### Hotspot extraction and construction of locus sets

We extract sub-sets of genomic entities (SNPs, CpGs and genes) on which we perform network inference based on the *trans* -meQTL reported by [12] (Supplementary Table 9 of their study) and the *trans* -eQTL obtained from the eQTLGen consortium [9, 60]. For GTEx, we obtained current (GTEx v8) tissue specific *trans* -eQTL from [61].

#### Hotspot extraction

The list of *trans*-meQTL results obtained from [12] was already pruned for independent genetic loci and was used as provided in the paper supplement. To remove redundant highly correlated genetic loci, we pruned the eQTLGen *trans*-eQTL by selecting the eQTLs with (1) the highest minor allele frequency and (2) the largest number of trans genes for each LD cluster (1 Mbp window, $R^2 > 0.2$). For GTEx, we merged eQTL by combining SNPs with $R^2 > 0.2$ and distance < 1 Mbp to independent genetic loci and kept all *trans* genes (genes associated with eQTL genotype) of the individual SNPs for this locus. The SNP with the highest MAF was selected as a representative SNP for the hotspot. We defined hotspots as genetic loci with $\ge 5$ *trans* associations, yielding 107 hotspots for the meQTL and 444 for the eQTLGen data. For GTEx, this approach yielded the single *trans* hotspot in skeletal muscle tissue presented in this paper. The only other tissue in which a *trans* hotspot could have been defined was testis tissue. However, as prior data for this tissue were not readily available, we decided to continue solely with the skeletal muscle hotspot. In [12], the authors provide a total of 114 meQTL hotspots per our definition. We discarded 7 of the 114 meQTL hotspots (SNPs rs10870226, rs1570038, rs17420384, rs2295981, rs2685252, rs57743634, rs7924137, as either no *cis* genes are available or no gene expression data were measured for any of the annotated *cis* genes (mostly lincRNAs, miRNAs and pseudogenes; Additional File 1: Table S1), which are needed for locus set definition (see below). All hotspots and the corresponding *trans* meQTLs and eQTLs are listed in Additional File 2: Table S1 and S2.

#### Locus sets

To mitigate the $N< <P$ problem in network inference [6], where the number of features or parameters far exceeds the number of samples, we run the inference on a subset of genomic entities (SNPs, genes and CpGs) induced by *trans* hotspots. We therefore gathered all genes, which could be involved in mediating the observed QTL effects and thus were considered during the network inference, in the form of *locus sets* for each hotspot. We bridge the gap between the involved chromosomes by including transcription factor binding site (TFBS) information collected from *ReMap* [62, 63] and *ENCODE* [64–66] as well as human protein-protein interaction (PPI) information available via *“theBioGrid” *[67, 68] (version 3.5.166). We filtered *ReMap* and *ENCODE* TFBS for blood related cell types by selecting all samples which contain at least one of the following terms: “amlpz12_leukemic,” “aplpz74_leukemia,” “bcell,” “bjab,” “bl41,” “blood,” “lcl,” “erythroid,” “gm,” “hbp,” “k562,” “kasumi,” “lymphoblastoid,” “mm1s,” “p493,” “plasma,” “sem,” “thp1,” and “u937.” Genes in the PPI network were filtered for genes expressed in whole blood (GTEx v6p [69] $RPKM > 0.1$). We enumerated all entities to be included in the locus set by performing the following steps:

1. Define set $S_L$ for a locus *L* and add the QTL entities (QTL SNP $\mathcal {S}$ and *trans*-QTL genes/CpGs $\mathcal {T} = \{T_1,\dots ,T_q\}$, where *q* is the number of associated *trans* entities for *L*)
2. Add all genes encoded within 500kb (1Mbp window) of $\mathcal {S}$ as **SNP-Genes** to $S_L$ (set $\mathcal {G}_C$)
3. For meQTL hotspots, add genes in the vicinity of each $\mathcal {T}_i \in \mathcal {T}$ (previous, next, and overlapping genes with respect to the location of $\mathcal {T}_i$) as **CpG-Genes** to $S_L$ (set $\mathcal {G}_T$)
4. Add all **TFs** with binding sites within 50bp of each CpG or binding in the promoter region of each gene over all $\mathcal {T}_i \in \mathcal {T}$ to $S_L$ (set $\mathcal {G}_{TF}$)
5. Add shortest path genes $G_{SP}$, i.e., genes which connect $\mathcal {G}_{C}$ (step 2) with $\mathcal {G}_{TF}$ (step 4) according to BioGrid PPIs to $S_L$

To define $G_{SP}$, we added only genes which reside on the shortest path between the *trans* entities $\mathcal {T}$ and the SNP-Genes $\mathcal {G}_{C}$ in the induced PPI sub-network, i.e., containing all genes and their connections which can be linked to either $\mathcal {G}_{C}$ or the TFs $\mathcal {G}_{TF}$. Specifically, we added the CpGs to the filtered BioGrid PPI network [67], connected them to the TFs ($\mathcal {G}_{TF}$) which show binding sites in their vicinity and calculated node weights based on network propagation as described in [12]. We then extracted nodes on paths with maximal total propagation score based on node-wise propagation scores *PS*. For this, we weighted node scores proportional to $(-1) \times PS$ and then calculate the minimal node-weight paths between *trans* entities $\mathcal {T}$ and SNP-Genes $\mathcal {G}_{C}$ using the *sp.between()* method of the *RBGL* R package (version 1.56.0, R interface to the Boost Graph Library [70]) and extracted all genes on the resulting shortest paths. All nodes of the generated locus set were subsequently used as inputs to the network inference.

### Prior generation

We utilized several data sources to define priors for possible edges between and within different omics levels. Each possible edge between entities in the locus set can only be assigned a single type of prior. Specifically, the different priors include:

- SNP-to-Gene priors, for edges between the SNP $\mathcal {S}$ and SNP-Genes $\mathcal {G}_C$
- Gene-to-Gene priors, for edges between all gene-gene combinations except TFs $\mathcal {G}_{TF}$ and their eQTL based targets in $\mathcal {T}$
- CpG-to-Gene priors, for edges between CpGs in $\mathcal {T}$ and their neighboring genes $\mathcal {G}_T$
- TF-to-target priors, for edges between TFs $\mathcal {G}_{TF}$ and their targets in the *trans* set $\mathcal {T}$

#### SNP-to-Gene

To obtain SNP-to-Gene edge priors, we downloaded the full GTEx v6p whole-blood eQTL table [71]) and calculated, for each SNP-Gene pair, the local false discovery rate (lFDR, [72]) using the *fdrtool* R package (version 1.2.15). As described in Efron et al. (2008) [72], the lFDR represents the Bayesian posterior probability of having a null case (i.e., that the null hypothesis is true) given a test statistic. We therefore defined the prior for a specific SNP $\mathcal {S}$ and a SNP-Gene $\mathcal {G_C}$ as $p_{\mathcal {S}\mathcal {G_C}} = 1-lFDR_{\mathcal {S}\mathcal {G_C}}$.

#### Gene-to-Gene

We formulate *Gene-to-Gene* edge priors by combining public GTEx v6 gene expression data [44] with PPI information from the BioGrid [67] to retrieve co-expression *p*-values and the respective lFDR for pairs of genes connected by a protein–protein interaction. A special case are priors between TFs and their target genes as identified via ChIP-seq (see above), which are not considered as *Gene-to-Gene* edges but are handled separately as described under “TF-to-target priors” below. GTEx v6p RNA-seq gene expression data were downloaded from the GTEx data portal [69]. Expression data for GTEx were filtered for high quality samples (RIN $\ge 6$) and log2 transformed, quantile normalized, and transferred to standard normal distribution before removing the first 10 principle components to remove potential confounding effects [73]. Priors were derived for all Gene-Gene pairs with PPIs in the BioGRID [67] network, where a gene $\mathcal {G} \in \mathcal {G}_C \cup \mathcal {G}_{TF}$ (for meQTL) or $\mathcal {G} \in \mathcal {G}_C \cup \mathcal {G}_{TF} \cup \mathcal {T}$ (for eQTL). For each pair, we calculated the Pearson correlation *p*-values in the GTEx expression data and subsequently determined the lFDR over all *p*-values. The prior for two genes $\mathcal {G}_A$ and $\mathcal {G}_B$ was then set to $p_{\mathcal {G}_A\mathcal {G}_B} = 1-lFDR_{\mathcal {G}_A\mathcal {G}_B}$.

#### CpG-to-Gene

For the *CpG-to-Gene* priors (meQTL context only), we utilized two strategies, distinguishing between TF-CpG priors (i.e., priors between CpGs and TFs showing binding sites near the CpG site, described below under “TF-to-target priors”) and CpG-to-Gene priors (i.e., where the gene itself is encoded near the CpG). For the *CpG-to-Gene* priors, we utilized the genome-wide chromHMM [74] states (15 states model) identified in the Roadmap Epigenomics project [46, 75]. These states reflect functional chromatin states in 200bp windows and were obtained using histone mark combinations as identified via ChIP-sequencing. We quantified a CpGs potential to affect a nearby gene, $p_{T_x}$, by retrieving the proportion of Roadmap cell-lines in which the CpG resides within a transcription start site (TSS) related state (see Table 1). We further adjusted the $p_{T_x}$ by weighting state information according to the Houseman blood cell type estimates available from our data. To this end, we took the population mean for each of the Houseman cell proportion estimates and multiplied them with the chromHMM state proportions. A specific CpG-to-Gene prior for a CpG $\mathcal {T}_i \in \mathcal {T}$ and a gene $\mathcal {G}_{T_i} \in \mathcal {G}_T$ was then set to $p_{\mathcal {T}_i\mathcal {G}_{T_i}} = p_{T_x}$, if the genomic distance $d(\mathcal {T}_i, \mathcal {G}_T) <= 200bp$.

**Table 1:** Description of chromHMM states used in our analyses as given at https://egg2.wustl.edu/roadmap/web_portal/chr_state_learning.html. Boldfaced states were defined as “active transcription” states and used to set CpG-Gene priors

State no.	Mnemonic	Description
**1**	**TssA**	**Active TSS**
**2**	**TssAFlnk**	**Flanking active TSS**
3	TxFlnk	Transcr. at gene 5′ and 3′
4	Tx	Strong transcription
5	TxWk	Weak transcription
6	EnhG	Genic enhancers
7	Enh	Enhancers
8	ZNF/Rpts	ZNF genes and repeats
9	Het	Heterochromatin
**10**	**TssBiv**	**Bivalent/poised TSS**
**11**	**BivFlnk**	**Flanking bivalent TSS/Enh**
12	EnhBiv	Bivalent enhancer
13	ReprPC	Repressed polycomb
14	ReprPCWk	Weak repressed polycomb
15	Quies	Quiescent/low

#### TF-to-target priors

We formulate separate priors for all edges between transcription factors $\mathcal {G}_{TF}$ and *trans* CpGs (meQTL) and *trans* genes (eQTL) in $\mathcal {T}$. Priors were only set for TF-to-CpG edges were we observe a TF binding site (from ReMap/ENCODE, see above) within 50bp of the CpG. For TF-to-Gene edges, we only considered pairs were the TF has a binding site 2000 bp upstream and 1000 downstream of the gene’s TSS. In both cases, if the TFBS criteria are met, we set a fixed large prior of 0.99 for all $\mathcal {G}_{TF}$-$\mathcal {T}$ pairs to represent the strong protein-DNA interaction evidence of ChIP-seq data. TFBS for skeletal muscle tissue were predicted using factorNet (see the “TFBS prediction for muscle tissue” section).

Finally, the priors for all remaining possible edges which were not set based on one of the criteria described above, i.e., Gene-Gene pairs without PPI evidence, TF-CpG pairs without ChIP-seq evidence, and SNP-Gene pairs without eQTL in the GTEx data, were set to a small pseudo-prior $p_{pseudo} = 1\times 10^{-7}$.

### Ground truth network generation, data simulation, and prior randomization

We performed a simulation experiment for each of the meQTL hotspots. For each SNP $\mathcal {S}$ and its corresponding locus set $\mathcal {S}_L$, we first collect the corresponding prior matrix $\mathcal {P}_S$ with priors defined as described above. We generate 10 erroneous ($\mathcal {G}_N$) ground truth graphs $\mathcal {G}_N^{10}, \mathcal {G}_N^{20} \dots \mathcal {G}_N^{100}$ by switching edges in the graph while keeping the degree distribution of a sampled graph $\mathcal {G}_T$. $\mathcal {G}_T$ is generated using all entities of $\mathcal {S}_L$ by uniformly sampling from $\mathcal {P}_S$, i.e., $\mathcal {G}_T$ contains an edge $e_{ij}$ for each element $p_{ij}$ of $\mathcal {P}_S$, if $p_{ij} > p_{pseudo}$ and $runif(0,1) <= p_{ij}$, where *runif*(0, 1) generates uniformly distributed random numbers between [0,1]. This procedure effectively introduces errors in the study. For instance, by switching 10% of the edges from $\mathcal {G}_T$ to generate $\mathcal {G}_N^{10}$, and making sure, that the new edges are not present as priors in $\mathcal {P}_S$, we introduce a error degree of 10% when comparing $\mathcal {P}_S$ to $\mathcal {G}_N^{10}$. We simulate data for each $\mathcal {G}_S \in \{\mathcal {G}_T, \mathcal {G}_N^i$; $i \in \{10, 20, \dots , 100\}\}$ using the *bdgraph.sim()* method of the *BDgraph* package with parameters: *p *= |$\mathcal {S}_L$| (number of nodes), graph = $\mathcal {G}_S$, *N *= 612 (number of samples in LOLIPOP), and mean = 0. This approach generates normally distributed data with a covariance structure as defined by the ground truth graph. We want to assess the impact of having discrete (genotype) data present for the network inference. To this end, we converted the SNP variable in the simulated data to genotype dosages (0,1,2) reflecting the allele frequencies of the genetic variant used in this simulation run. Specifically, we transformed the Gaussian data obtained from *bdgraph.sim()* to discrete values using the frequencies of the individual dosages for the SNP in the LOLIPOP data as quantile cut points. For each of these simulated data individually, we infer the network models and compare the inferred networks to the respective ground truth graphs $\mathcal {G}_T, \mathcal {G}_N^{10}, \dots , \mathcal {G}_N^{100}$. We added one additional comparison, evaluating a prior on the density of the observed graph. For this, we estimated a single prior value reflecting the desired density for all edges based on a binomial model. We use the number of edges $|E_{\mathcal {G}_T}|$ of all sampled graphs $\mathcal {G}_T$ for a single run, the total number of possible edges $|E_T| = (N * (N-1)) / 2$, with *N* the total number of available nodes, and set the prior as

$$p_{rbinom} = max(\frac{1}{N_S}~*~\frac{\sum _{\mathcal {G}_T}{|E_{\mathcal {G}_T}|}}{|E_T|},~p_{pseudo}),$$

where $N_S$ is the number of sampled graphs (i.e., the number of randomizations). For each hotspot, we repeated the above simulation procedure 100 times to obtain stable results. We repeated the simulation analysis for different scenarios based on available sample size, including (1) prior error analysis for low sample size (*N *= 70) and (2) a no-error scenario with different sample sizes. For the latter, we sub-sampled the simulated data to retain only 50, 100, 150, $\dots$ 600 samples and performed network inference on these data. Finally, we investigated the effect of “prior completeness” on inference performance. For this, we progressively removed 10%, 20% $\dots$ 90% of the priors for the inference (replacing prior values for selected edges with our pseudo prior) and repeated the inference for the adjusted priors.

### Network inference

Based on the data and priors gathered for the individual hotspots, we set out to infer the regulatory networks which are best supported by these data. We evaluated several state-of-the art methods with respect to their applicability to this problem, both in a simulation study (see above) and via replication of inferred networks in real-world data from two large human population-based cohorts. We applied *GeneNet* [48, 49], the graphical lasso (*glasso*, [35]), *BDgraph* [34], and *iRafNet* [38] as well as *GENIE3* [50] on the individual data to reconstruct regulatory networks using the respective *CRAN* [76] and *bioconductor* [77] R packages. An overview on the used inference methods and package versions is given in Additional File 1: Table S2. Methods were chosen to reflect a range of different approaches (i.e., shrinkage based partial correlation in *GeneNet*, Bayesian MCMC sampling in *BDgraph*, lasso in *gLASSO* and tree-based inference in *iRafNet* and *GENIE*3), based on whether or not implementation was readily available and whether prior knowledge could be incorporated. The well-known *GeneNet* and *GENIE*3 methods are not capable of utilizing prior information but were used as a reference for comparison to the other methods. We performed parameter optimization for all methods. For instance, for the graphical lasso, we implement screening of the penalty parameter lambda based on cross validation (details below). Lambda translates to a global weight for the edge-wise prior information supplied to the model. We hence effectively screen different weights for the priors for all methods. To obtain final networks, we use the same strategy suggested in [78] for GENIE3, GeneNet, iRafNet, and BDgraph (see below) to obtain optimal edge cutoff points. For the graphical lasso, we used an approach based on cross validation (also described below) to screen the penalty parameter.

#### GENIE3

To infer networks with GENIE3, we again used the NA filtered data (see above) with the *GENIE3* method of the package followed by the *getLinkList* method using default parameters. GENIE3 generates a ranked list of regulatory links which do not relate to any statistical measure and hence a cutoff for the link weights has to be identified manually [79]. To define an optimal cutoff, we first divide the list of weights into 200 quantiles (marking 200 distinct cutoffs) if the number of unique link weights exceeded 200. We then extracted for each cutoff the respective regulatory network and compared it to a scale free topology analogously to the approach used in [78], generating $R^2$ values indicating the goodness-of-fit to the topology. To choose the final network, we followed the approach suggested by Zhang et al. (2005) [78], which suggests to use networks with $R^2 > 0.8$. If none of our networks fit that criteria, we choose the network with the highest $R^2$. Cutoffs have been similarly obtained for the other methods described below.

#### GeneNet

For the application of GeneNet, we first filtered any CpG probes from the data containing missing values. We then estimated the regulatory network by calling first the *ggm.estimate.pcor* followed by the *network.test.edges* and *extract.network* methods, all with default parameters.

#### BDgraph

We used BDgraph to infer networks under consideration of prior information as well as without prior information (*BDgraph* and $BDgraph_P$) using the *bdgraph* method of the *BDgraph* CRAN package (version 2.61). The following parameters were set: *method = “gcgm”*, *iter = 10000*, *burnin = 5000*. We further set the *g.prior* parameter to the prior matrix collected for the hotspots and the *g.start* parameter to the incidence matrix obtained from the prior matrix by setting all entries with prior information $>0.5$ to 1 and all others to 0. For comparison with the no prior case, we kept all parameters the same but omitted the *g.start* and *g.prior* parameters. The graph was then obtained from the fitted model using the *select* method of the package with parameter *cut = 0.9*, thereby only choosing edges with a posterior probability of at least 0.9.

#### iRafNet

We use *iRafNet* to infer networks using prior information (it is not possible to run it without specifying priors). We called the *iRafNet* method of the package, setting the parameters *ntrees = 1000*, *mtry = round(sqrt(ncol(data)-1))*, and *npermut = 5* using the data filtered for missing values (see above) and then used the *Run_permutation* method with the same parameters. The final network was extracted using the *iRafNet_network* method by supplying the output of the previous method calls and setting the FDR cutoff parameter *TH = 0.05*. We used a custom implementation of *iRafNet* adjusted to make use of multiple CPUs which we made available at https://github.com/jhawe/irafnet_custom.

##### glasso

Similar to BDgraph, we utilized the graphical lasso both with and without prior information. To infer the graphical lasso models, we used the *glasso* method available in the *glasso* CRAN package and set the parameter *penalize.diagonal = FALSE*. The *glasso* takes a regularization parameter $\lambda$, which implies either strong penalization of edges (high $\lambda$) or weak penalization (low $\lambda$) of parameters. This parameter can also be supplied as a matrix $\Lambda$ of size $n \times n$ (where *n* is the number of nodes/variables) in order to supply individual parameters for individual edges. We integrated the prior information by first transforming the prior matrix $\mathcal {P}$ such that $\Lambda = 1~-~\mathcal {P}$ and then supplying $\Lambda$ as the regularization matrix containing values for each possible edge. This approach is similar to what has been proposed in [36, 37]. In addition, we screened a selection of penalization factors $\omega$ for both the prior and the none prior case to construct the optimal graphical lasso network with respect to the Bayesian Information Criterion (BIC). For the prior case, we included $\omega$ in the model by setting $\Lambda = \Lambda \times \omega$). For the non-prior case, we set $\lambda = \omega$. We performed 5-fold cross validation and inferred the model for all $\omega \in \{0.01, 0.015, ..., 1\}$ on the training set (containing 80% of the data) and then selected the $\omega$ yielding the minimal mean BIC value on the test data over all folds to generate the final network.

### Method evaluation via simulation study and cross cohort replication

To identify the inference method best suited for our application, we evaluated all described network inference methods independently on the simulated data as to (1) their ability to reconstruct the underlying ground truth network as well as (2) their robustness to errors in the supplied prior information. We further compared networks inferred independently on the different cohort data to assess stability of the network inference across different, yet similar, data. Performance was measured in terms of Matthews correlation coefficient (MCC) [34, 80, 81] between the inferred networks and the respective ground truth (simulation study) and the inferred networks on the different cohorts (cross cohort replication). It is defined as:

1 $$\begin{aligned} MCC = \frac{TP\times TN - FP \times FN}{\sqrt{(TP+FP)\times (TP+FN)\times (TN+FP)\times (TN+FN)}} \end{aligned}$$

MCC was calculated using the *compare()* method as implemented in the *BDgraph* package (version 2.61).

### Transcription factor activities

We calculated transcription factor activities for all TFs extracted from the ReMap/ENCODE (see above) using the *plsgenomics* R package’s *TFA*.*estimate*() method (version 1.5-2) [82]. As input, we used the full expression matrix from KORA and LOLIPOP (whole-blood) and from GTEx (skeletal muscle) individually to obtain tissue specific TFAs. TFBS information was encoded as an incidence matrix indicating for each TF its target genes. Target genes were defined as genes with an TFBS within their promoter region (2000bp upstream and 1000bp downstream of the TSS).

### Network prioritization and final network creation

Networks were inferred for each of the 107 meQTL and 444 eQTLGen *trans* hotspots with $gLASSO_P$ and $BDgraph_P$, yielding networks with a median number of 67 and 20 edges for $gLASSO_P$ and 72 and 27 for $BDgraph_P$ over all hotspots, respectively. We filtered and ranked the networks based on the following criteria.

#### GWAS filtering

We filtered genetic loci with hits in genome-wide association studies (GWAS) using the current version (v1.0.2) of the GWAS catalog [83]. We extracted high LD (> 0.8) SNPs and SNP aliases using the SNiPA tool [84] for each hotspot SNP. If any of the extracted SNP rsIDs had a match in the GWAS catalog, the hotspot’s inferred network was permitted for downstream analysis.

#### Network ranking

We utilized a self-devised graph score for prioritizing final models for further investigation. The graph score reflects desirable biological properties, which can be assumed for the networks underlying the *trans* -QTL hotspots. The score is formulated such that (1) the adjacency of SNP-genes and SNPs is rated positively, (2) the presence of trans entities is rated positively if they are not connected directly to the SNP, and (3) high graph density is rated negatively (i.e., sparser graphs yield higher scores). Specifically, the graph score $S_G$ for an inferred graph *G* is defined as:

$$\begin{aligned} S_{G} = -log10(D_{G}) * [\frac{1}{|\mathcal {G_{C}}|}(\sum _{i=1}^{|G_{S}|}{1} - \sum _{i=1}^{|\overline{G_{S}}|}{1}) + \frac{1}{|\mathcal {T}|}(\sum _{i=1}^{|G_{T}|}{1} - \sum _{i=1}^{|\overline{G_{T}}|}{1})] \end{aligned}$$

where $D_G$ is the graph density, $\mathcal {G_C}$ is the set of all SNP-Genes, $\mathcal {T}$ is the set of all *trans* entities, $G_S$ is the set of all SNP-genes adjacent to the SNP in *G* or directly connected to another SNP-Gene, $\overline{G_S}$ is the set of SNP-Genes in *G* but not connected directly to the SNP or one of the other SNP-Genes, $G_T$ is the set of trans entities in *G* which can be reached from any SNP-Gene without traversing the SNP or another *trans* gene first, and $\overline{G_T}$ is the set of *trans* genes directly connected to the SNP. Only the cluster containing the SNP, i.e., the SNP itself and any nodes reachable from the SNP via any path in *G*, is considered for calculating $S_G$; if the SNP is not present or no SNP gene has been selected in the final graph, the score is set to 0.

In addition to the graph score, we ranked networks according to the total number of edges and nodes to prioritize smaller networks for detailed analysis.

#### Graph merging

Finally, we constructed hotspot networks containing only high confidence edges by merging the individually obtained networks from the two cohorts (KORA and LOLIPOP) and keeping only edges and nodes present in both networks. Nodes without any adjacent edges are not included in the final graph.

### Priors for skeletal muscle tissue

We downloaded Muscle tissue eQTL generated by Scott et al. (2016) [85] from https://theparkerlab.med.umich.edu/data/papers/doi/10.1038/ncomms11764/ and used local FDRs calculated from the provided p-values to define SNP-Gene priors. Gene expression data for muscle tissue were obtained from the ARCHS^4^ [47] database. We downloaded all relevant muscle expression data using the keywords “Skeletal_Muscle” with the ARCHS4 loader [86] (*N *= 194 samples). Expression data were normalized using the *ComBat* method implemented in the *sva* R package while providing dataset series ID as the batch parameter.

#### TFBS prediction for muscle tissue

We used *factorNet* [87] to predict transcription factor binding sites from DNAse-seq chromatin accessibility data obtained from muscle cell lines. First, we trained a *factorNet* model for all TFs available for the K562 cell-line in ReMap [62]. ReMap ChIP-seq peaks functioned as a ground truth during training, DNAse-seq data from ENCODE (dataset accession ENCFF971AHO) [64, 65, 88] and DNA sequence information formed the inputs. We downloaded DNAse-seq data for the LHCN-M2 muscle cell-line from ENCODE in bigWig format for hg38 (dataset accession ENCFF639MPM [89]). *FactorNet* was then run with default parameters, using as input (1) the DNA sequence and (2) the bigWig DNAse track for each of the trained ChIP-seq tanscription factors (*N* = 179 TFs from ReMap). High confidence TFBS were extracted by setting a *factorNet* score cutoff of 0.999, merging overlapping regions and then retaining only regions with a $width < W_{0.95}$, where $W_{0.95}$ is the 95th percent quantile of the widths of all obtained regions.

### Colocalization analysis

GWAS summary statistics for schizophrenia were identified using the GWAS Atlas [90] and downloaded from [91]. Whole-blood *trans*-eQTL summary statistics for all SNP-Gene pairs from eQTLgen were downloaded from the eQTLgen website [60] (file “Full trans-eQTL summary statistics”). We used *fastENLOC* [92, 93] to calculate colocalization probabilities as described in the *fastENLOC* Github README using default parameters. To generate probabilistic eQTL annotations, we used *DAP-G* [94, 95] and created PIP files as needed using *TORUS* [96]. For LD block definition, we utilized data available from LDetect [97, 98].

## Results

### Trans-QTL hotspots define regulatory network candidates

In this study, we aimed to reconstruct regulatory networks to explain *trans* quantitative trait locus (*trans*-QTL) hotspots on a molecular level through simultaneous integration of multi-omics data [6]. We sought to improve our understanding of likely disease associated *trans*-QTL hotspots [10, 15] to reveal their mechanisms of action and gain insights into regulatory pathways and ultimately into disease processes.

**Fig. 1:**
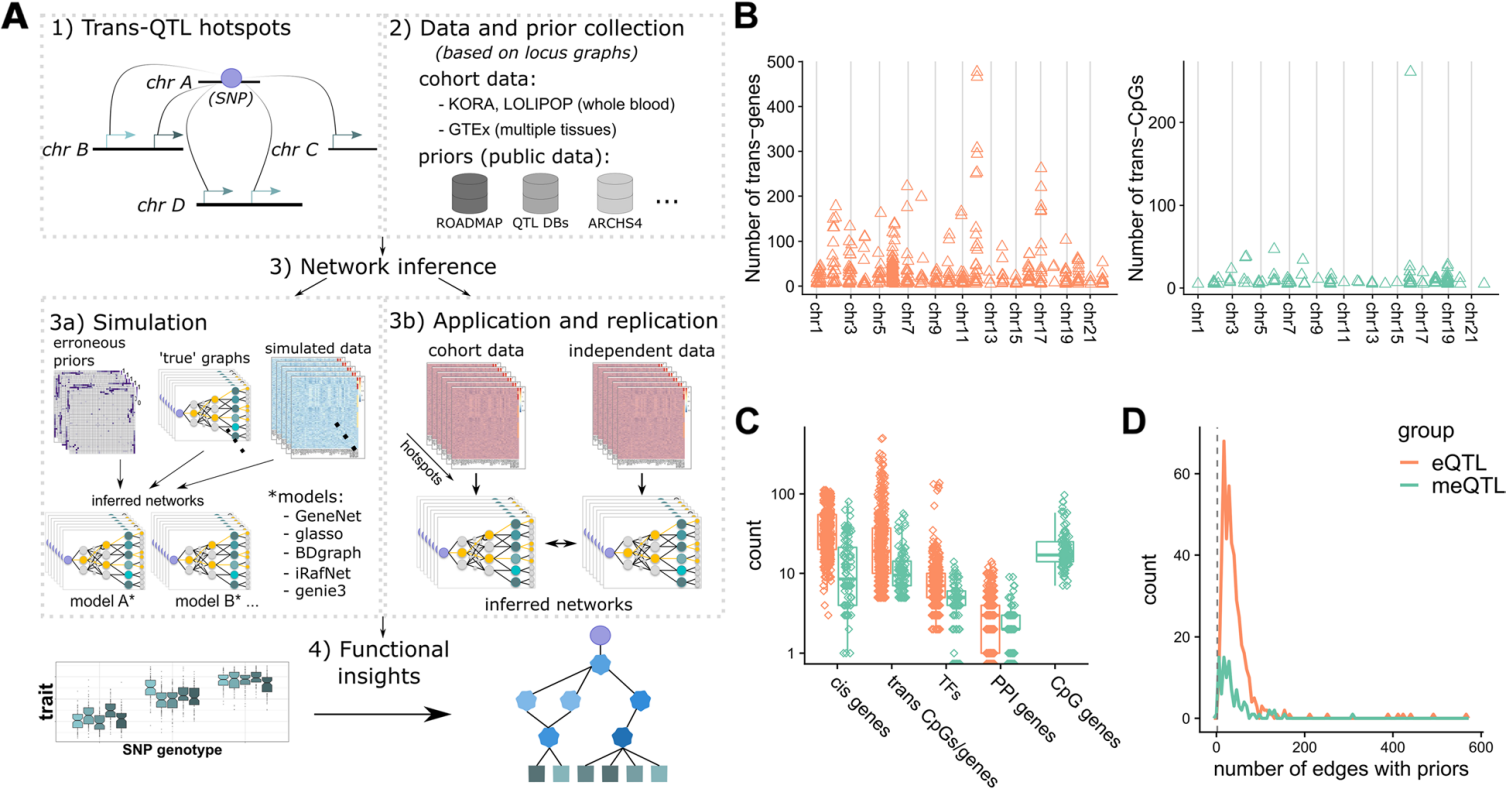
Project overview. **A** A graphical abstract of the analyses performed in this project. **B** A global view on the collected eQTL (orange) and meQTL (green) hotspots. The *x*-axis indicates ordered chromosomal positions for *trans* genes and *trans* CpG sites, respectively. **C** The total number of different genomic entities gathered over all hotspots during locus set creation (log scale). **D** Density plots of the number of possible network edges with available prior information (*x*-axis) over all hotspots, zoomed in to area between 0 and 1000. Same color coding is used in **B**–**D**

Our general analysis strategy is depicted in Fig. 1A and consists of the following steps: (1) curate QTL hotspots, (2) gather individual level molecular data and independent prior information, (3a+b) benchmark network inference methods in simulation (a) and replication (b) study to select the optimal method, and (4) infer and interpret networks identified in the cohort data. The individual level molecular cohort data used for network inference include gene expression and DNA methylation (whole blood, KORA, and LOLIPOP cohorts) or gene expression data (skeletal muscle, GTEx) in addition to the genotype data available in all cohorts.

We obtained *trans* hotspots from the methylation QTL (meQTL) discovered in whole-blood in the KORA [54] and LOLIPOP [56] cohorts reported by Hawe et al. [12] and the expression QTL (eQTL) published by the eQTLGen consortium [9], yielding a total of 107 and 444 *trans* -loci per QTL type, respectively (Fig. 1B, see Section 2). In addition to the whole-blood derived hotspots, we curated a single *trans*-eQTL hotspot in Skeletal Muscle tissue from GTEx v8 [44, 45, 59] which was analyzed separately.

For each hotspot, we aimed to identify the causal gene at the genetic locus affected by the SNP and the intermediate genes that give rise to the observed *trans* associations. To this end, we collected sets of candidate genes with different roles for each locus. We term these sets “locus sets” (see Section 2). A locus set contains the SNP defining the hotspot and the respective *trans-*associated traits, i.e., “*trans* CpGs” for meQTL and “*trans* genes” for eQTL. We further add genes encoded near the SNP as candidate causal genes (“*cis* genes”) and genes in vicinity of the CpGs of the meQTLs (“CpG genes”). Finally, as potential intermediate genes, we include transcription factors binding near the *trans* associated genes/CpGs (“TFs”) as well as genes residing on the shortest path between *trans* CpGs/*trans* genes and *cis* genes in a protein-protein interaction (PPI) network (“PPI genes”). *Cis* genes form potential candidate regulator genes of the locus and the inclusion of the PPI genes and TFs allows us to bridge the inter-chromosomal gap between the SNP and the *trans* CpGs/*trans* genes. An overview of entities collected over all loci for both QTL types is given in Fig. 1C.

One main aspect of this work is the use of any form of biological prior information, including continuous scores, to guide network inference. We hence collect prior information for all possible edges between entities contained in locus sets in addition to the individual level molecular data (Fig. 1A—step 2). In total, four distinct types of edges are annotated with prior information: *SNP-Gene*, *Gene-Gene*, *TF-CpG*/*TF-Gene*, and *CpG-Gene* edges. All prior information is generated from tissue matched, public data independent of the data used during network inference (see Section 2).

Figure 1D indicates the total number of edges annotated with prior information over all hotspots. For meQTL and eQTL, a minimum of 2 and 3 edges per hotspot show prior evidence, respectively, and most hotspots get only relatively few priors compared to the total number of possible edges (median 26 and 94, respectively). However, several networks collect priors for over 100 edges (8 and 209 loci with $>=100$ priors for meQTL and eQTL). As expected, the total number of edges with prior information per locus correlates with the total number of possible edges in the respective loci. However, the fraction of all possible edges annotated with prior information decreases (Additional File 1: Fig. S1).

### Benchmark of network inference methods

#### Simulation study shows benefit of data-driven priors

Numerous methods for regulatory network inference have been proposed (e.g., [35, 48, 50], see also [6]). We therefore sought to select the method best suited for this study before investigating individual hotspots in detail (see Fig. 1A—step 3). To this end, we performed an extensive simulation study (Fig. 1A—step 3a) to evaluate the performance of five distinct methods (see Additional File 1: Table S2 for a method overview) in reconstructing ground truth graphs from simulated data and prior information.

To ensure that the simulated ground truth has the same characteristics as the real data, we randomly sampled graphs from the prior distributions for each of the observed 107 meQTL hotspots (median number of nodes: 28). In a second step, we sampled the quantitative individual level data corresponding to the ground truth graph for 612 individuals. A total of 100 simulation runs were performed for each hotspot. To study the impact of errors in the prior, we randomly rewired a fraction of edges (0 to 100%, see Section 2) in the ground truth graph that we used for comparing the networks inferred on simulated data. We further sub-sampled decreasing numbers of individuals from the full simulated data to assess the effect of sample size, and we sub-sampled the prior edges used for analysis to assess the effect of incomplete priors.

**Fig. 2:**
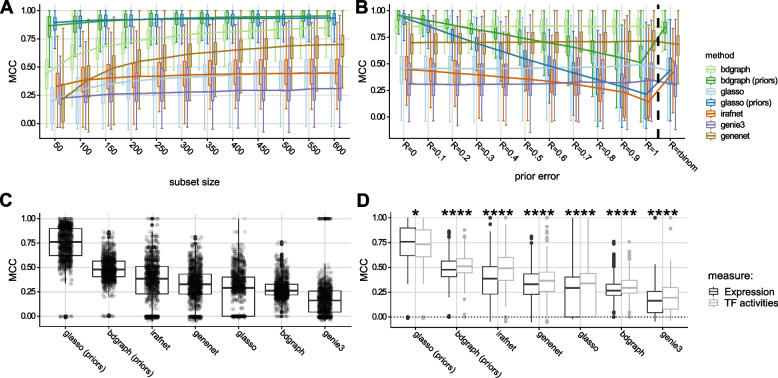
Method comparison results. **A** Results of simulation study: y-axis shows the Matthews correlation coefficient (MCC) as compared to the simulated ground truth; *x*-axis indicates increasing sample size from left to right; colors indicate different inference methods. **B** Similar to **A**, but *x*-axis indicates increasing errors in the prior matrix from left to right for *N *= 612 samples. Group (‘*rbinom*’) indicates uniform prior set to reflect degree distribution of true graph. **C** MCC (*y*-axis) between networks inferred on KORA and LOLIPOP data for same locus for all methods (*x*-axis). **D** contrasts MCC across cohorts using TF expression (dark gray) versus using substituted TFAs (light gray). Boxplots show medians (horizontal line) and first and third quartiles (lower/upper box borders). Whiskers show $1.5 * IQR$ (inter-quartile range); for **B**, dots depict individual results, and for **C**, stars indicate significant difference between expression/TFA results for each method (Wilcoxon test, *: $P \le 0.05$, **: $P \le 0.01$, ***: $P \le 0.001$, ****: $P \le 0.0001$)

We gauge the relative gain in performance attributable to prior information for both *gLASSO* and *BDgraph* by always training two distinct models, one with priors ($gLASSO_P$, $BDgraph_P$) and one without priors (*gLASSO*, *BDgraph*). The implementation of *iRafNet* always requires a prior matrix, whereas both *GeneNet* and *GENIE*3 cannot utilize prior information and hence were trained only with the simulated data. We focused on Matthews correlation coefficient (MCC) [80] as a balanced performance measure to compare inferred networks to the respective ground truth (see also [34]). Figures 2A and 2B show the results for the simulation study for all methods (see also Additional File 1: Tables S3–S8 and Fig. S2).

Both $gLASSO_P$ and $BDgraph_P$ exhibit improved performance with relatively low standard deviation in terms of MCC as compared to their non-prior counterparts in low and high sample size settings . $BDgraph_P$ and $gLASSO_P$ exhibit the best performance across all methods on simulated data that we deem close to our real-world scenario (low prior error at 20% and high subset size at N=600). In general, the performance of most methods is affected by low sample sizes with *BDgraph* showing slightly better performance than all other methods. Moreover, both $gLASSO_P$ and $BDgraph_P$ outperform all other methods as long as the prior error does not exceed 10% ($gLASSO_P$) and 30% of incorrect edges in the prior graph, in which case *BDgraph* achieves the highest median MCC over all methods. Prior information containing less than 30% of incorrect edges significantly improves the network reconstruction with BDgraph, while glasso can profit from priors with even more errors, with up to 60% of wrong edges (Additional File 1: Fig. S3A). When the prior contains a very high fraction of incorrect edges (80%), increasing sample sizes become more important (Additional File 1: Figs. S2, S4). In general, a good method should be able to dynamically adjust the overall weight given to the prior information depending on the quality of the prior and the sample size during cross validation. We verified this for the glasso as an example. The overall weight for the prior (rho) indeed decreases with increasing sample size and error in the prior (Additional File 1: Fig. S12). *GeneNet* performs well in all simulations, whereas *GENIE*3, *gLASSO*, and *iRafNet* show about average performance with *iRafNet* achieving worst results overall. Overall, methods including prior information significantly outperform the other methods at 10% incorrect prior edges (Additional File 1: Fig. S3B). In addition to the curated prior matrices, we also generated a prior matrix reflecting the sparsity of the true graph (column “rbinom” in Fig. 2B and Additional File 1: Tables S3 and S4, see Section 2). Our results indicate that information about sparsity of the underlying network already improves network inference performance. We note that results are similar when looking at sensitivity instead of MCC, while the specificity of individual methods only changes slightly for different fractions of incorrect prior edges (Additional File 1: Fig. S5 and Tables S5 and S8) due to the class imbalance towards absent edges. Current prior networks are expected to be incomplete [99, 100] and different reference networks showed limited overlap (Additional File 1: Table S9). Therefore, we complemented our analysis of prior error with an analysis of “prior completeness” by assessing how performance changes when keeping only *F *= 10%, 20%, $\dots$, 90% of prior information (edges in the prior network). Even when keeping only 10% of prior information, $gLASSO_P$ achieves better performance than *gLASSO* (Additional File 1: Fig. S6, *P *< 2.2e−16, two-sided Wilcoxon test) and, as expected, the difference in performance increases the more prior information is available. Bdgraph seems more sensitive to incomplete prior information and $BDgraph_P$ only outperforms *BDgraph* when keeping at least 60% of the original prior information. Further, prior based methods and specifically $BDgraph_P$ outperform non-prior methods in the task of identifying the correct *cis*-gene by recovering associations between the discrete SNP and continuous gene expression data types (Additional File 1: Fig. S7), when using independent eQTL data as prior. Direct comparison of the impact of prior completeness and error in the prior showed that errors in prior information are more harmful for prediction performance than incomplete priors (see Additional File 1: Fig. S8). This is in line with the expectation that wrong prior edges increase the chance of predicting both false-positive and false-negative edges, while missing prior edges only increase the chance of not predicting edges that are actually present (false negatives). Finally, we investigated run-time requirements for the individual methods. Here, *GeneNet* outperformed all other methods, followed by *gLASSO*, *BDgraph*, and *GENIE*3, with *iRafNet* exhibiting slowest run-time (Additional file 1: Fig. S9).

#### Inferred networks replicate in independent datasets

In addition to the simulation study, we evaluated the methods on real world data from two large population cohorts: the KORA (Cooperative Health Research in the Region of Augsburg) [54] and LOLIPOP (London Life Sciences Population) [55] cohorts (see Fig. 1A2 and Section 2). Data from both cohorts were generated from whole-blood samples and contain imputed genotypes as well as microarray measurements of gene expression and DNA methylation for a total of 683 (KORA) and 612 (LOLIPOP) samples. Since for these data no ground truth is available, we evaluate robustness of the networks inferred by the individual methods via cross cohort replication. For each hotspot, we collected data for all genes, CpGs, and the SNP in the locus set for KORA and LOLIPOP and separately inferred networks in both cohorts for all models. Obtained networks were then compared between cohorts using MCC to get a quantitative estimate of how robust the network inference is across different datasets for the same hotspot yielding scores for KORA versus LOLIPOP and vice versa (i.e., one network functioning as the reference). The results of this analysis are shown in Fig. 2C. With respect to MCC, models supplied with prior information ($gLASSO_P$, $gLASSO_P$ and *iRafNet*) show the best performance with $gLASSO_P$ coming up as the most robust method followed by $gLASSO_P$ and *iRafNet*. Noticeably, of the top methods $gLASSO_P$ shows much less variance compared to $gLASSO_P$ and *iRafNet*. Ignoring prior information lead to a drop in performance for both *gLASSO* and *BDgraph*, which leads to *GeneNet* outperforming both methods. Finally, *GENIE*3 shows worst performance in this setting.

Replication between cohorts can either be driven by strong evidence in the data in both cohorts or by strong priors. To assess the contribution of the evidence in the data, we grouped inferred edges according to their replication status and prior availability. The evidence in the data was quantified by the correlation in the replication data set. For both *gLASSO* and *BDgraph*, we observed that around half of the inferred edges are mostly driven by the prior and the replicated edges without prior show stronger evidence in the data (Additional File 1: Fig. S10).

#### Transcription factor activities as a proxy to TF activation

Transcription factor activities (TFAs) estimated from transcription factor binding sites (TFBS) and gene expression data have been suggested as an alternative to using TF gene expression in inference tasks [101], since a transcription factor’s expression level alone might not reflect the actual activity of a TF (driven for instance by its phosphorylation state). To evaluate whether TFAs could improve our inference, we estimated TFAs for all TFs based on their expression and ChIP-seq derived TFBS from ReMap [62] and ENCODE [64, 65] (see Section 2). We applied the same cross cohort replication strategy as above and compared MCCs from the TFA based analysis to the previous results using a one-sided Wilcoxon test. Figure 2D shows the results of TFA (light gray boxes) versus gene expression (dark gray boxes)-based analysis in terms of MCC for all available hotspots. For all models but $gLASSO_P$, TFAs yield a significantly higher MCC (Wilcoxon test $P<0.05$) as compared to using the original TF expression data (see also Additional File 1: Table S10).

Detailed investigation of real world data was therefore focused on networks obtained from $gLASSO_P$ and $BDgraph_P$ and TF expression was substituted by TFA estimates for all subsequent analyses.

### Replication of previous findings

Before seeking new mechanistic insights and generating novel hypotheses from *trans*-QTL hotspots we first checked whether our approach can replicate previous findings. Hawe et al. [12] inferred gene regulatory networks from *trans*-meQTL hotspots using a two-step inference approach, whereas our approach simultaneously integrates all functional data relying on PPI and ChIP-seq information as prior knowledge. We thereby avoid the need for post-hoc correlation testing of e.g., SNP-gene and CpG-gene edges. For the comparison, we extracted three of their reported networks and evaluated the overlap with the networks inferred in this study.

Overall, the comparisons indicate relatively strong concordance between the two approaches with MCCs of 0.52, 0.8, and 0.66 (see Additional File 1: Table S11 for details). Moreover, for all three networks, our simultaneous inference approach yielded more edges and nodes than the two-step approach (39%, 34%, and 22% novel edges and 7%, 12%, and 47% additional nodes for rs9859077, rs730775, and rs7783715, respectively), which might have been missed by the two-step approach as it relies on known PPI and ChIP-seq information. Although the total number of inferred edges with ChIP-seq prior information is relatively similar to the total number of edges without PPI and ChIP-seq prior evidence (e.g., 155 vs 106 for the rs9859077 locus), replication is much higher for edges with ChIP-seq evidence (e.g., 84% vs 5% for the rs9859077 locus). This is a likely consequence of the choice of priors: a fixed high value for ChIP-seq evidence, while neighboring genes without PPI evidence only received the small pseudo prior (see Section 2).

**Fig. 3:**
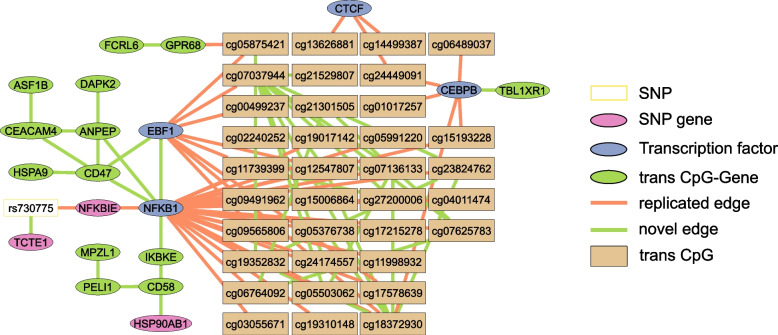
Comparison of the random walk based network reported in [12] and the network inferred from functional omics data in this study for the rs730775 locus. Shown is the complete network constructed from the omics data, edge color indicates replication/novelty. Orange edges: replicated with respect to the random walk network. Green edges: novel in our network. White box: SNP; pink nodes: SNP-genes; blue nodes: TFs; brown boxes: CpGS; green nodes: CpG-genes

Figure 3 contrasts the two networks obtained for the *rs730775* hotspot using (1) the two-step approach by Hawe et al. [12] and (2) the network inferred in this study using $gLASSO_P$. Orange edges show replicated and green edges indicate novel edges. In Hawe et al. [12], the authors described a regulatory network involving the *rs730775* SNP connected via *NFKBIE* to *NFKB1* which connects to the *trans*-CpG sites. This main pathway is also discovered in our approach (i.e., *rs730775* $\leftrightarrow$ *NFKBIE* $\leftrightarrow$ *NFKB1* $\leftrightarrow$ *CpG sites*) in addition to some of the initially reported TFs (blue nodes). Of these, *NFKB1* is connected to most of the *trans* CpGs (69%, 24 out of 35) as was the case in the original network. However, we also identify patterns of CpG genes (green nodes) connected to the TFs which were not previously identified. Overall, the integrated approach using prior information leads to good replication of previous networks and includes novel connections leading to potential new insights in target gene regulation.

### A trans regulatory network for a schizophrenia susceptibility locus

In order to demonstrate the effectiveness of our approach in getting mechanistic insights from *trans* -QTL associations, we inferred networks for all meQTL [12] and eQTL [9] hotspots using whole blood data from the KORA and LOLIPOP cohorts and the prior based $gLASSO_P$ and $BDgraph_P$ models (see Section 2, all networks are listed in Additional File 2: Table S3). Based on the GWAS catalog (v1.0.2, [83]), graph properties, and a custom graph score (see Section 2), we prioritized a *trans* acting locus that has previously been associated with schizophrenia (SCZ).

**Fig. 4:**
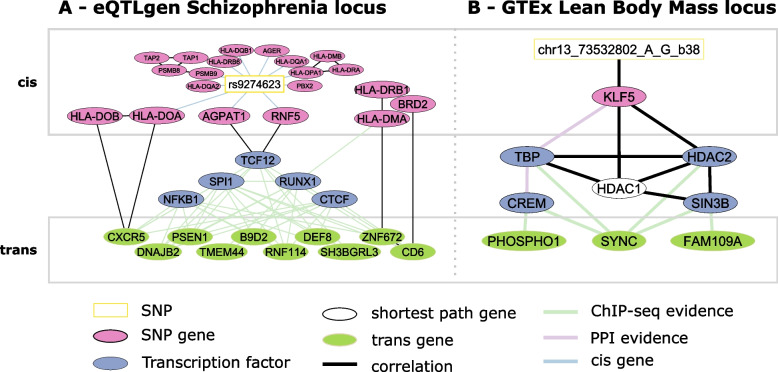
Inferred networks for the schizophrenia susceptibility locus rs9274623 obtained from eQTLgen (**A**) and the rs9318186 locus obtained from GTEx (**B**). The white boxes indicate sentinel SNPs, pink ovals indicate SNP-Genes, blue ovals transcription factors, and white ones shortest path-derived genes. Light green ovals represent genes trans-associated to the SNP. Black edges were inferred during network inference. In addition to being inferred, colored edges indicate ChIP-seq protein-DNA binding evidence (green), protein-protein interaction in the BioGrid (purple), and whether or not a gene is encoded in *cis* of the linked entity (blue)

The network involves the *trans*-eQTL locus around the *rs9469210* (alias *rs9274623* according to [84]) SNP in the human leukocyte antigen (HLA) region on chromosome 6 shown in Fig. 4A.

*rs9274623* has been associated with SCZ [102] and is a *cis*-eQTL for several of its directly connected SNP-genes (e.g., *PBX2*, *RNF5*, and *HLA-DQA1*) in the eQTLGen study. The network inference prioritized the two genes *RNF5* and *AGPAT1*, which connect the genetic locus to the associated trans genes. *RNF5* showed differential expression for SCZ cases vs controls in addition to its expression being associated with an additional independent SCZ susceptibility SNP (rs3132947, $R^2=0.14$ in 1000 genomes Europeans) located in the HLA locus [103]. *AGPAT1* is involved in regulation of phospholipids [104], the dysregulation of which has been implicated in schizophrenia before [105]. In addition, several genetic variants in *AGPAT1* intronic regions have previously been associated with SCZ [106–108]. *TCF12* is a paralog of *TCF4* and *TCF3* which are known E-box transcription factors and are expressed in multiple brain regions [109]. *TCF4* loss-of-function mutations are the cause of Pitt-Hopkins syndrome (a syndrome causing intellectual disability and behavioral changes amongst other symptoms) [110], and regulatory SNPs relating to *TCF4* have been associated with SCZ [111, 112]. The transcription factor *SPI1* (*PU.1*) is linked to Alzheimer’s disease likely by impacting neuroinflammatory response [113] and was found to interact with its network neighbor *RUNX1* in modulating gene expression [114]. Moreover, *RUNX1* has been implicated in rheumatoid arthritis, a disease negatively associated with SCZ and which hence might share susceptibility genes with SCZ [115, 116]. Interestingly, several genes encoded in the HLA locus, which has been implicated in SCZ and other psychiatric and neurological disorders [117–120], were picked up by our inference downstream of the identified transcription factors (*HLA-DOA*, *HLA-DOB*, *HLA-DRB1*, *HLA-DMA*, and *BRD2*). The *NFKB1* pathway, represented in the network through *NFKB1*, has further been recognized as an important regulatory and developmental factor of neural processes and was found to be dysregulated in patients with SCZ [121]. Finally, 10 of the 40 discovered *trans* genes of the locus are connected to the SNP via the selected TFs. Of these, *SH3BGRL3* [122] has already been linked to SCZ and *PSEN1* [123], *B9D2* [124], and *CXCR5* [125] as well as *DNAJB2* [126] and were implicated in other neurological disorders. In addition, the *trans* gene *RNF114* has previously been shown to play a role in the *NFKB1* pathway [127]. A formal colocalization analysis using fastENLOC [93] showed evidence of a common causal variant underlying the SCZ GWAS signal [128] and each of the *trans* -eQTL of *TMEM44*, *PSEN1*, *DNAJB2*, and *CD6* (SNP-level colocalization probability of 0.95, 0.92, 0.87, and 0.42, respectively; see Section 2 and Additional File 1: Fig. S11).

Our approach highlighted a potential regulatory pathway involving diverse genes related to SCZ and other neurological disorders. While some of the genes were not previously reported in this specific disease context (e.g., *CD6*, *BRD2*, *DEF8*), their association to this network indicates a potential role in SCZ pathogenesis. The colocalization analysis further hints at a potential causal relationship between these genes and SCZ.

### Application to GTEx skeletal muscle tissue

All above analyses were focused on whole-blood data. However, the proposed strategy can be applied to data from any biological context. To demonstrate this, we investigated the recently published *trans* -eQTLs from the GTEx v8 release [44, 59, 61]. We pinpoint a single LD block in Skeletal Muscle tissue, but not in other tissues, which is a *trans*-eQTL hotspot according to our definition (see Section 2) and for which we inferred regulatory networks. Since we ca not use the GTEx derived prior information to infer networks in GTEx tissue, we set out to curate muscle tissue specific priors from independent datasets. We utilized muscle tissue based eQTL from Scott et al. (2016) [85] and gene expression data curated from the ARCHS^4^ [47] database and generated tissue specific TFBS using factorNet [87] on DNAse-seq data obtained from ENCODE [64–66] (see Section 2). The resulting network for the $gLASSO_P$ model is shown in Fig. 4B.

The genetic variant rs9318186 is a *cis*-eQTL of *KLF5* in GTEx v8 skeletal muscle ($P=6.1x10^{-37}$), and a proxy of it ($R^2=0.88$) has been associated with *lean body mass* (LBM). *KLF5* itself, too, has been associated with LBM in a transcriptome-wide association study integrating GWAS results with gene expression [129] and with lipid metabolism in *KLF5* knockout mice [130]. In addition, several other genes in the network have been associated with related phenotypes: both *HDAC1* and *HDAC2* have been found to control skeletal muscle homeostasis in mice [131], work together with *SIN3B* in the SIN3 core complex to regulate gene expression, and are involved in muscle development [132]. TATA-binding protein (*TBP*) is a well-known transcription factor and important for the transcriptional regulation of many eukaryotic genes [133]. The *trans* gene *SYNC* was found to interact with dystrobrevin (*DMD* gene) in order to maintain muscle function (during contraction) in mice as well as being associated with neuromuscular disease [134, 135]. In addition, in Seim et al. (2018) [136], the authors investigated the relationship between obesity and cancer subtypes and found that *PHETA1*/*FAM109A* expression is associated with body mass index (BMI) in esophageal carcinoma in data from The Cancer Genome Atlas (TCGA). *PHOSPHO1* has been found to be involved in metabolism, specifically in energy homeostasis [137] and has also been associated via DNA methylation with BMI [138] and HDL levels, which in turn have been negatively associated with LBM [139]. Dayeh et al. (2016) [140] further showed decreased DNA methylation at the *PHOSPHO1* locus in skeletal muscle of diabetic vs. non-diabetic samples. The remaining gene in the network (*CREM*) has not yet been described in the broader context of LBM but a GWAS meta-analysis executed by Wang et al. (2014) [141] hinted at association of a *CREM* SNP (rs1531550, $P=1.88x10^{-6}$) with elite sprinter status. These results suggest that *KLF5* may exert its specific functions through transcriptional regulation via the SIN3 core complex including *TBP*, with a potential involvement of *CREM*, of the *trans* genes *PHOSPHO1*, *SYNC*, and *PHETA1*/*FAM109A*.

## Discussion

In this study, we introduced a Bayesian framework for the inference of undirected regulatory networks underlying molecular *trans*-QTL hotspots across multi-omics data types using existing prior knowledge. We compiled a comprehensive set of context specific network edge priors from diverse biological databases and applied these in a multi-omics context on (1) simulated data and (2) real-world cohort data.

We followed recently published guidelines [142] to benchmark state-of-the-art network inference methods.

Based on the simulations, we observed that increasing the degree of error in the prior information significantly reduces method performance. Above 30% of incorrect prior edges, the performance for $BDgraph_P$ is inferior to the performance when not using the prior. This indicates that low levels of errors in edge priors still improve network inference, results which are in line with, e.g., Wang et al. (2013) [36], who used a modified graphical lasso approach, Christley et al. (2009) [33], who used an regularized ODE model and Greenfield et al. (2013) [32], who used a Bayesian regression framework. Both $BDgraph_P$ and $gLASSO_P$ outperform other methods, specifically also in recovering mixed edges between discrete SNP allele dosage and continuous gene expression levels. While $BDgraph_P$ shows overall better performance than $gLASSO_P$, the graphical lasso exhibits much lower run-time which can be an important practical consideration. In addition, prior based methods show better replication across different cohorts as compared to prior agnostic methods. While replication performance across cohorts might be driven by strong prior information (i.e., prior based methods tend to replicate prior information), we could show that replication is driven by both the functional genomics data and prior information (Additional File 1, Fig. S10). This shows that curated priors help to obtain more stable and confident results as compared to using functional data alone. Our simulation and replication results provide a comprehensive benchmark of established network inference methods and suggest that priors should be integrated in network inference tasks wherever possible.

Based on the benchmarking, we choose $BDgraph_P$ and $gLASSO_P$ for investigation of real-world cohort data. We reproduced several networks from a previous step-wise network inference approach [12]. Moreover, we were able to find additional connections that could not be detected by design of the previous approach, which only assessed established PPI and protein-DNA interactions. In contrast, our integrated approach considers all edges regardless of available prior evidence. Therefore, associations will emerge if the evidence in the functional data alone or in addition to the prior evidence is strong enough.

We highlight a novel regulatory network for the schizophrenia (SCZ) susceptibility HLA locus. The haplotype structure of the HLA locus warrants caution for the interpretation of the candidate genes based on *cis*-eQTL. Irrespective of the haplotype structure, our candidates *RNF5* and *AGPAT1* are defined by their connections in the network to the *trans* genes and therefore independently of the *cis-*eQTL. Expanding on similar previous observations based on *trans-*eQTL [9], the integrated network analysis including *trans-*eQTL genes suggests *RNF5* and *AGPAT1* as potential candidate genes which was not possible using *cis-*eQTL alone. Moreover, we observed strong evidence for colocalization of the genetic variants underlying the disease and the molecular traits. As the network for this locus was derived from whole-blood data, it is important to assess how these effects translate to tissues more relevant for schizophrenia. Generally, eQTL effects correlate strongly between blood and brain tissue [143] and consequently also networks building upon these genetic effects may translate between tissues.

To show that our approach can be applied across different tissues and technologies, we analyzed a skeletal muscle *trans*-eQTL hotspot from GTEx associated with lean body mass. The genes linked in the inferred network are overall coherent with the observed phenotype at this *trans*-acting locus (e.g., genes involved in lipid metabolism and skeletal muscle homeostasis [129–131, 135, 138]) and suggest involvement of novel genes.

Several practical considerations arise from our findings: first, a strong emphasis should be given to curating high quality continuous prior information from public biological data to keep error levels low. Our simulation results clearly demonstrated that prior information is only beneficial when the proportion of incorrect edges does not exceed 30% (Fig. 2B, Additional File 1: Fig. S3A). To ensure low error rates, one might consider using only experimentally validated protein-protein interactions or high-quality gene expression data to generate priors as the impact of missing edges is less detrimental than that of wrong edges (Additional File 1: Fig. S8). However, in line with literature observations [99, 100], the comparison of different reference networks showed relatively low overlap (Additional File 1: Table S9), which might lead to an incomplete or small set of priors. In this case, glasso might be considered the model of choice based on our prior completeness analysis (Additional File 1, Fig. S6). Next, the definition of hotspot locus sets and priors in this study mitigates the $N< <P$ problem. Using our approach, the total number of entities (variables) going into the network inference typically does not exceed the total number of available samples in our data. This benefit of the locus sets comes with the risk of missing certain genes needed to fully describe the *trans* effects. However, our strategy of curating a stringent set of relevant genes including transcription factors should enable most key regulator genes to enter the inference process and yields parsimonious and easily interpretable results. Finally, context (e.g., tissue) specific TFBS are not yet available for a large number of transcription factors. This potentially limits our approach to fewer applications. However, novel developments to predict TFBS from context specific open chromatin information (e.g., *factorNet* [87]) can help in carrying this strategy to more contexts as we showed for the GTEx skeletal muscle locus.

## Conclusions

This study describes a novel strategy for using comprehensive edge-wise priors from biological data to improve network inference for *trans*-QTL hotspots from human population scale multi-omics data. This facilitates the investigation of their underlying regulatory networks and enables the generation of novel mechanistic hypotheses for disease associated genetic loci. Moreover, we report a rigorous benchmark of state-of-the-art network inference methods for this task both in simulated and real-world data and highlight the benefit of including biological prior information to guide network inference.

## Supplementary Information

**Additional file 1** Supplementary material, supporting figures and tables.

**Additional file 2** Supplementary tables, large supporting tables with table titles as tabs.

## Data Availability

*Data*. The meQTL [12] and eQTL [9] associations used in this study are available via https://zenodo.org/record/5196216 and http://www.eqtlgen.org/, respectively. Raw genotype, methylation, and expression data from LOLIPOP analyzed during the current study are not publicly available due to data privacy reasons and the patient’s informed consent but are available from John Chambers (john.chambers@ntu.edu.sg) on reasonable request. Controlled data access to data of the KORA cohort can be obtained through https://helmholtz-muenchen.managed-otrs.com/. All other data used in this study (e.g., GTEx, Roadmap data) are publicly available. The lists of derived hotspots for both data sets are made available in the supplement of this paper. Trans-eQTLs of the eQTLgen consortium are available from https://eqtlgen.org/trans-eqtls.html[60]. The GTEx analysis v8 trans eQTLs of the GTEx consortium are available from https://storage.googleapis.com/gtex_analysis_v8/single_tissue_qtl_data/GTEx_Analysis_v8_trans_eGenes_fdr05.txt [61]. The gene expression data of the GTEx analysis v6p of the GTEx consortium are available from https://storage.googleapis.com/gtex_analysis_v6p/rna_seq_data/GTEx_Analysis_v6p_RNA-seq_RNA-SeQCv1.1.8_gene_rpkm.gct.gz [69]. The cis eQTLs of the GTEx analysis v6p of the GTEx consortium are available from https://storage.googleapis.com/gtex_analysis_v6p/single_tissue_eqtl_data/all_snp_gene_associations/Whole_Blood_Analysis.v6p.all_snpgene_pairs.txt.gz [71]. The transcription factor binding sites of the remap project are available from http://pedagogix-tagc.univ-mrs.fr/remap/download/remap2018/hg19/MACS/remap2018_all_macs2_hg19_v1_2.bed.gz [63]. ENCODE transcription factor binding sites are available from the ENCODE website http://hgdownload.cse.ucsc.edu/goldenPath/hg19/encodeDCC/wgEncodeRegTfbsClustered/wgEncodeRegTfbsClusteredWithCellsV3.bed.gz [66]. Data for the ENCODE DNAse1 experiment (accession ENCFF971AHO) are available from https://www.encodeproject.org/files/ENCFF971AHO/ [88]. Data from the ENCODE DNAse1 experiment (accession ENCFF639MPM) are available https://www.encodeproject.org/files/ENCFF639MPM/ [89]. BioGrid protein interaction data are available from https://downloads.thebiogrid.org/Download/BioGRID/Release-Archive/BIOGRID-3.5.166/BIOGRID-ORGANISM-3.5.166.tab2.zip [68]. Chromatin state annotations from the roadmap project are available from https://egg2.wustl.edu/roadmap/web_portal/chr_state_learning.html [75] Schizophrenia GWAS summary statistics are available from http://walters.psycm.cf.ac.uk/clozuk_pgc2.meta.sumstats.txt.gz [91]. Linkage disequilibrium data are available from the Ldetect bitbucket repository https://bitbucket.org/nygcresearch/ldetect-data/src/master/ [98]. *Code*. In case no other information is given above, all calculations were performed using standard Unix commands and version 3.5.2 of the R statistical computing language on a Centos 7 Unix system. The complete code and the software environment (Dockerfile) used in this project is provided via Github/Zenodo [144]. The workflows for both the cohort and the simulation studies were implemented in Snakemake [145] and are deposited alongside the code in the same Github repository.
