# Supplementary material for: Network reconstruction for trans acting genetic loci using multi-omics data and prior information
Source: Genome Med. 2022 Nov 7;14:125. doi: 10.1186/s13073-022-01124-9 (PMC9641770; doi:10.1186/s13073-022-01124-9)
Supplement: Supplementary file 1 — Additional file 1 Supplementary material, supporting figures and tables. [file 13073_2022_1124_MOESM1_ESM.pdf]

Supplementary Material for 'Network reconstruction for trans acting genetic loci using multi-omics data and prior information'

Johann S. Hawe, Ashis Saha, Melanie Waldenberger, Sonja Kunze, Simone Wahl, Martina Müller-Nurasyid, Holger Prokisch, Harald Grallert, Christian Herder, Annette Peters, Konstantin Strauch, Fabian J. Theis, Christian Gieger, John Chambers, Alexis Battle, and Matthias Heinig

Supplementary Figures

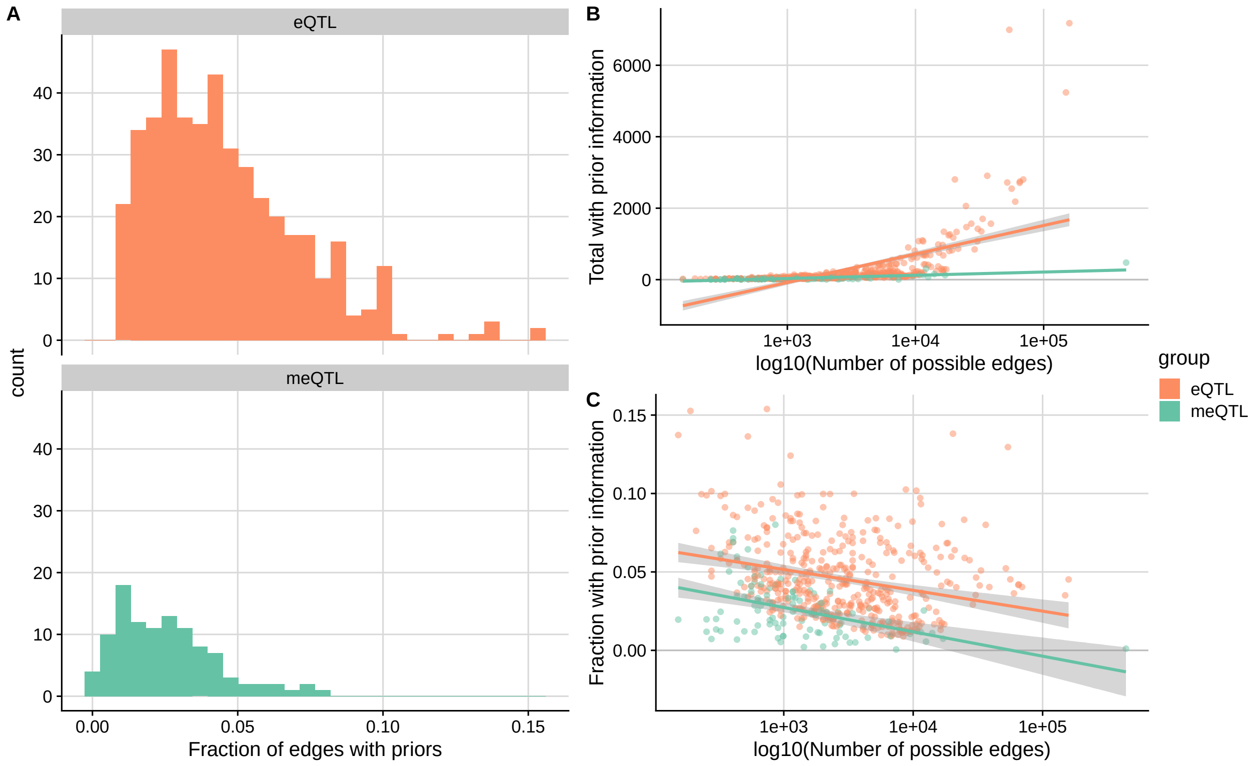

Figure S 1: Overview over the number of collected priors for all *trans* hotspots. A) Histograms of the fraction of edges with annotated prior information. B) Total number of collected priors per hotspot (y-axis) plotted against the total number of possible edges (x-axis, log-scale). The total number of edges with prior information increases as the edge space increases. C) Same as B), but showing the fraction of edges with prior information on the y-axis. The fraction decreases as the total number of possible edges increases. Colors indicated QTL type (orange: eQTL, green: meQTL). Fitted lines represent the line of best fit with a linear model, grey areas show standard errors.

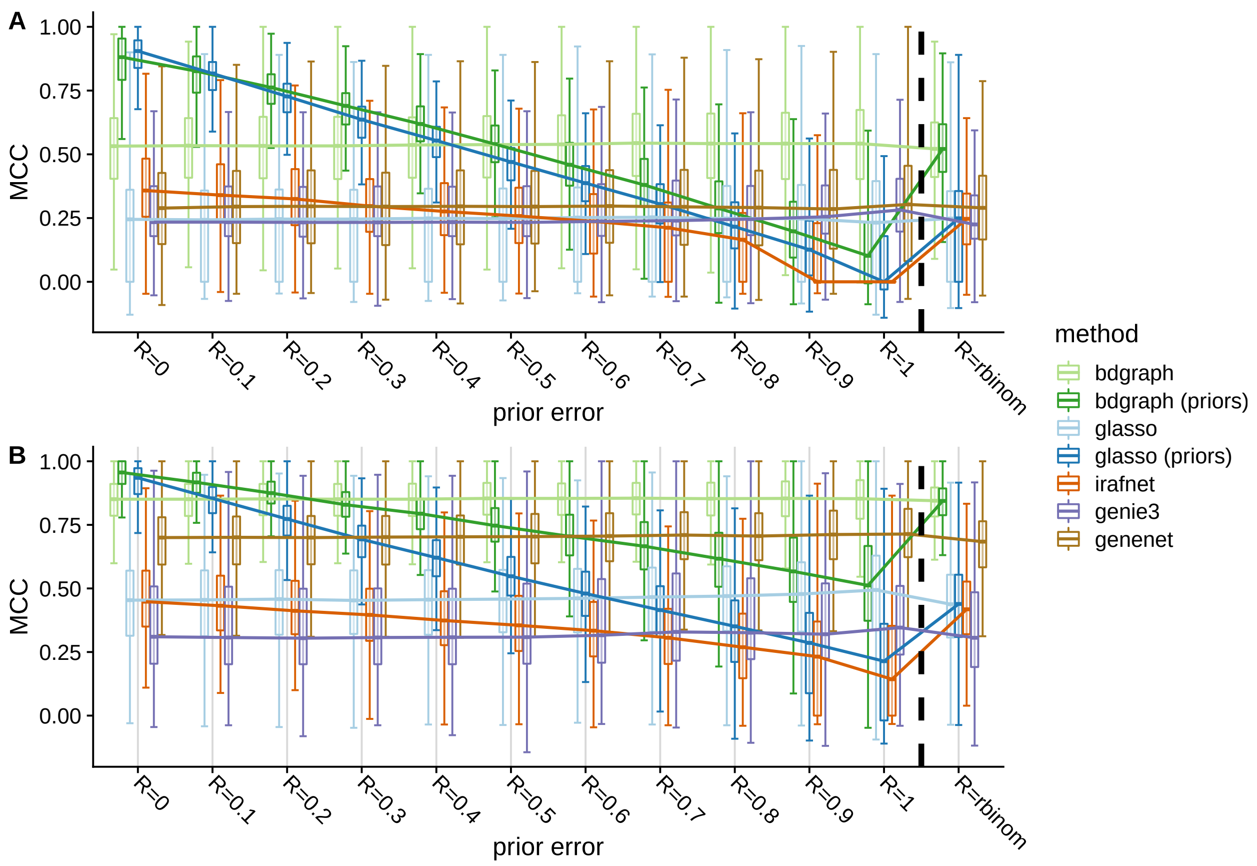

Figure S 2: Progression of MCC over all error degrees for a low sample size (A, N=70) and high sample size (B, N=612) setting. Y-axis shows the MCC, x-axis degree of prior error increasing from left to right. Colors indicate different methods. Boxplots show medians (horizontal line) and first and third quartiles (lower/upper box borders). Whiskers show 1.5*IQR (inter-quartile range).

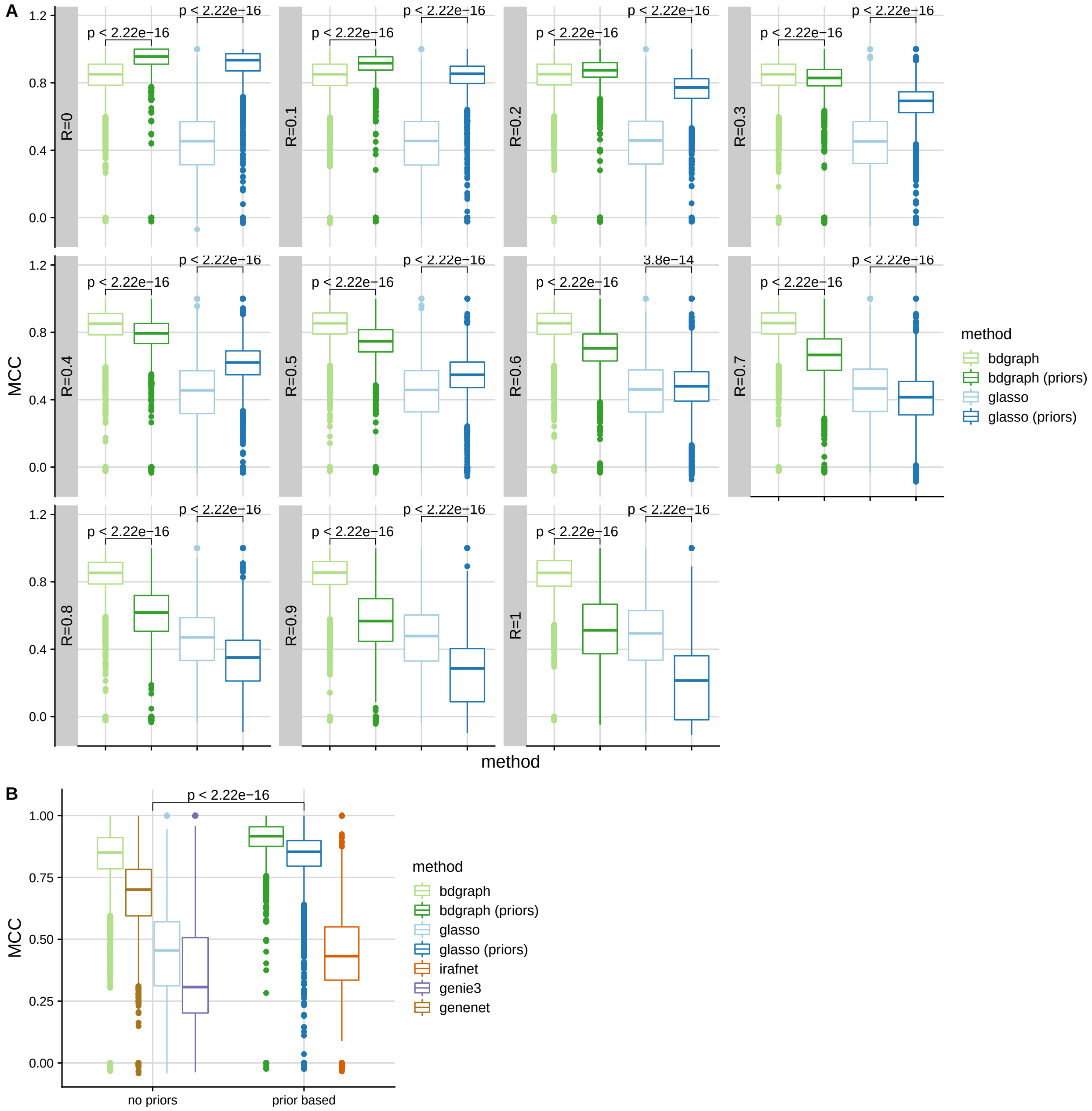

Figure S 3: Comparison of prior based vs non-prior based methods. Y-axis indicates MCC, x-axis different methods (A) and method categories (prior VS non-prior methods) (B). Colors highlight individual methods. A) Systematic comparison of prior and non-prior versions of BDgraph and glasso for varying degrees of error in the prior (R: fraction of wrong edges in the prior). P-values are computed using a two sided Wilcoxon test. B) Overall comparison of prior based and prior agnostic methods in terms of MCC in the simulation study with 10% error (R=0.1). Wilcoxon test shows significantly higher performance for prior based methods (P<2.22e-16).

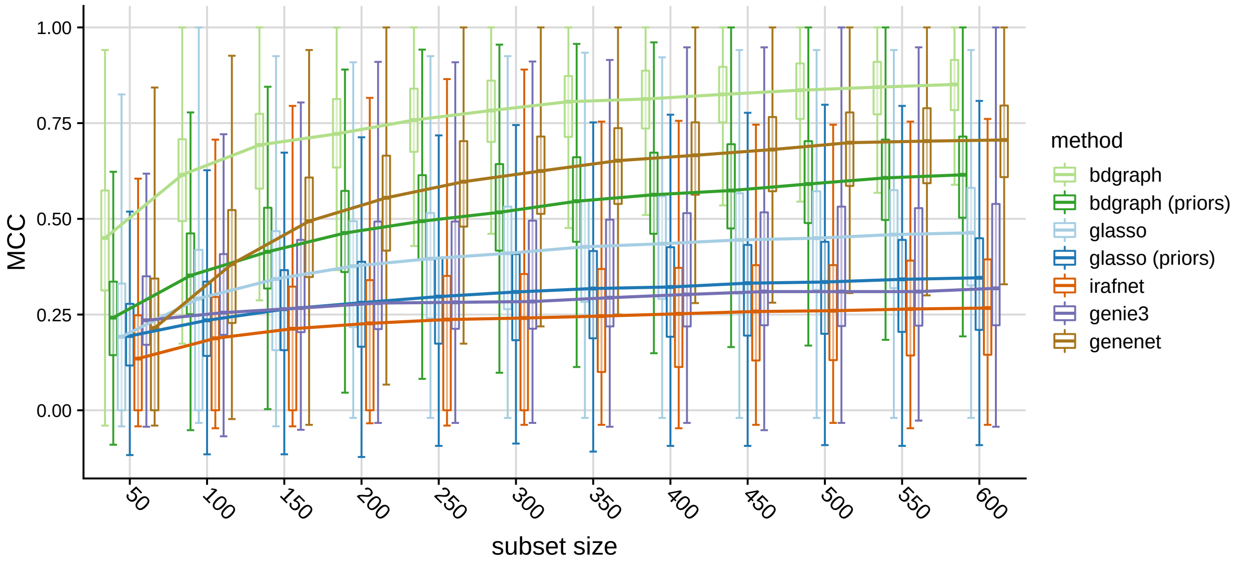

Figure S 4: Analysis of the effect of sample subset size on inference performance in a high prior error setting (R=0.8). Y-axis shows the MCC of inferred graphs VS the simulated ground truth, x-axis indicates the different sample sizes for inference increasing from left to right. Colors indicate the different models. 50 iterations of sampling were performed. Box-plots show medians (horizontal line) and first and third quartiles (lower/upper box borders). Whiskers show 1.5*IQR (inter-quartile range).

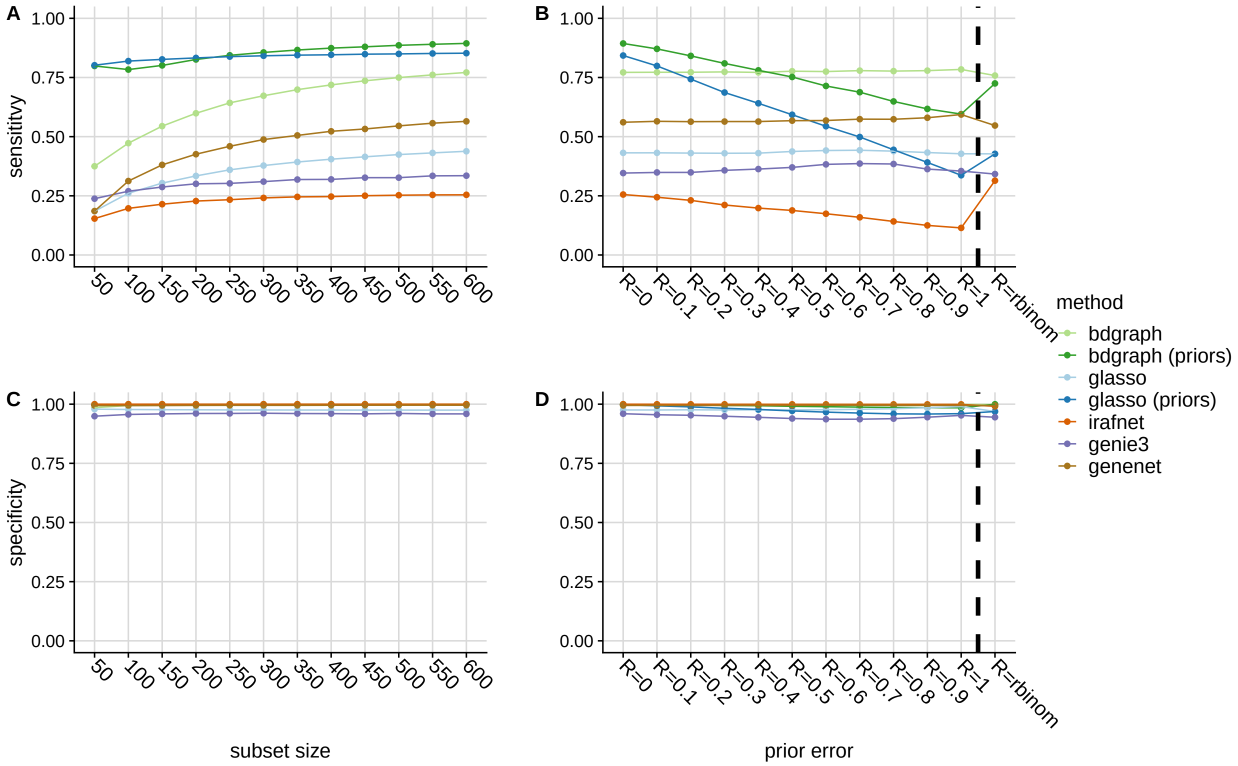

Figure S 5: Method comparison based on sensitivity (panels A and B) and specificity (panels C and D), similar to Figure **??** **A** and **B** where the MCC is shown. Colors indicate different methods, y-axis shows mean sensitivity/specificity across simulation runs. X-axes indicate increasing sample subset size (A,C) and increasing prior error (B,D) from left to right.

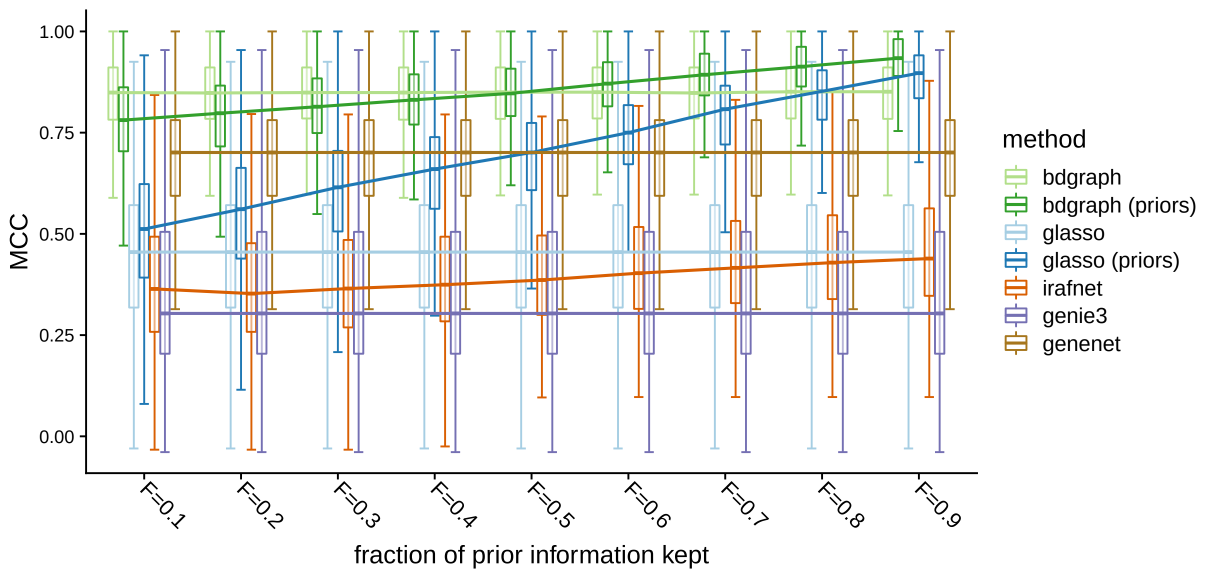

Figure S 6: Analysis of the effect of 'prior completeness' on inference performance. Y-axis shows the MCC of inferred graphs VS the simulated ground truth, x-axis indicates the fraction of prior information retained for inference, increasing from left to right. Colors indicate the different models. Boxplots show medians (horizontal line) and first and third quartiles (lower/upper box borders). Whiskers show 1.5*IQR (inter-quartile range).

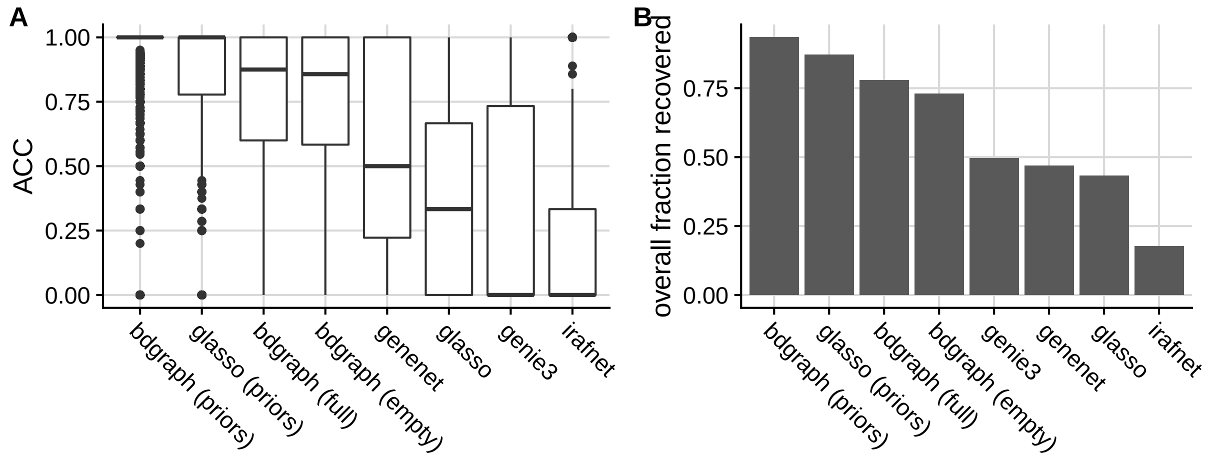

Figure S 7: Performance of inference methods in recovering SNP links (edges between discrete and continuous data types) in the simulation. A) Boxplots showing fraction of recovered SNP associations (y-axis) for each method (x-axis) over all simulations. B) Summarized fractions, where for each methods the total number of recovered SNP associations over the total number of SNP associations in the generated ground truth graphs is calculated. Boxplots show medians (horizontal line) and first and third quartiles (lower/upper box borders). Whiskers show $1.5 * IQR$ (inter-quartile range), dots indicate outliers.

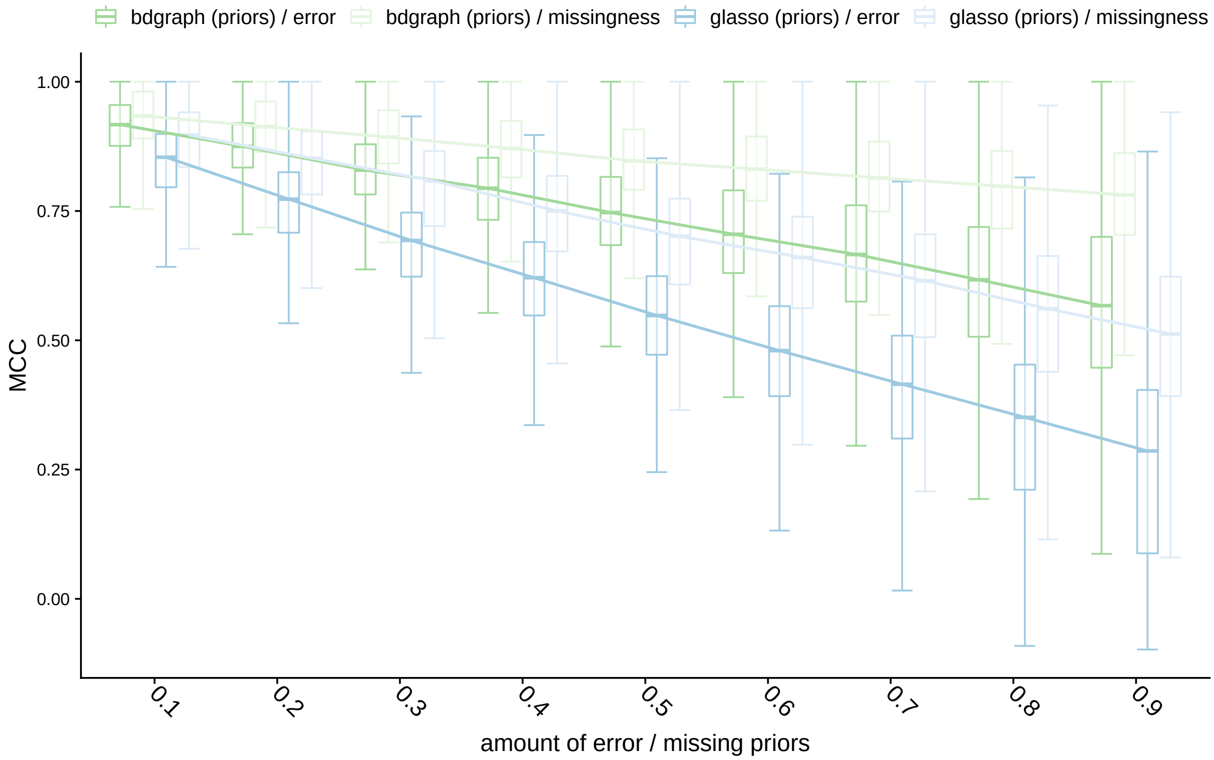

Figure S 8: Figure compares the effect of the fraction of missing edges in the prior (completeness) and the fraction of “wrong” edges in the prior (prior error). Both fractions are shown on the x-axis, while the Matthews correlation coefficient (MCC) is shown on the y-axis. The shading of the lines indicates which fraction is plotted: light shading corresponds to the fraction of missing and dark shading corresponds to the fraction of “wrong” edges in the prior used for analysis. The color indicates the type of model: green corresponds to $BDgraph_P$ and blue corresponds to $gLASSO_P$.

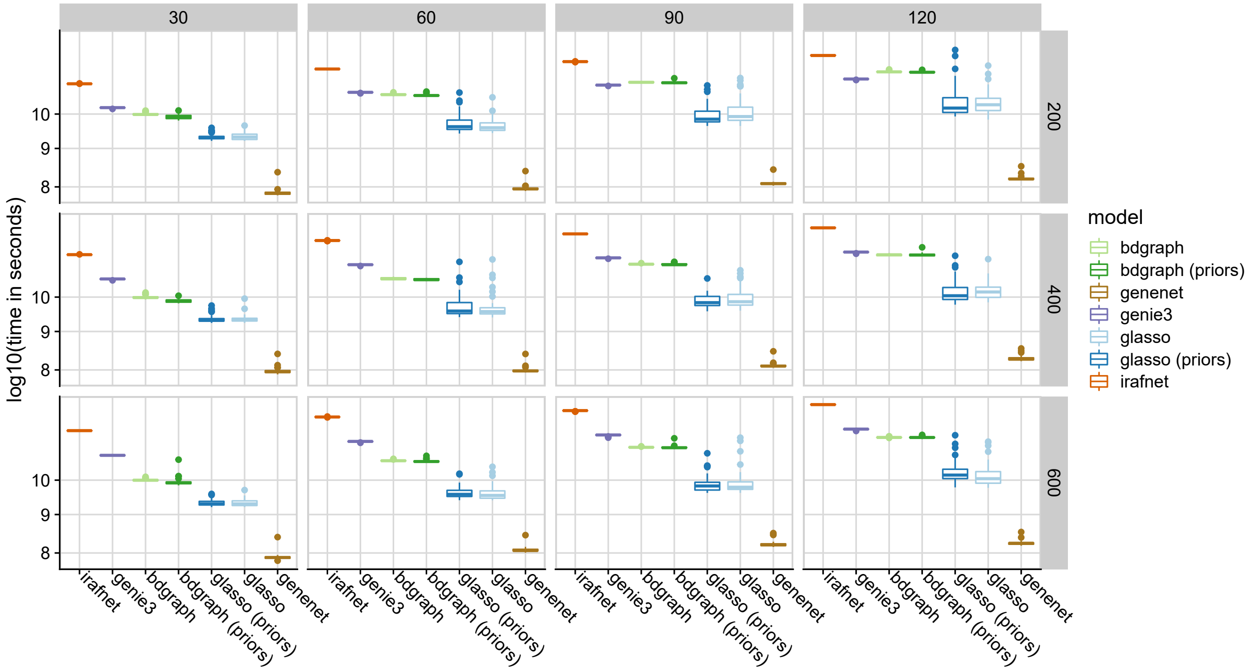

Figure S 9: Run-time performance of inference methods for simulated data with 50 iterations of random sampling. Rows indicate different sample sizes and columns the number of simulated input nodes. Y-axis indicates the log10 of the run-time for fitting the model in seconds. All calculations have been performed on a single machine using a single CPU core. Boxplots show medians (horizontal line) and first and third quartiles (lower/upper box borders). Whiskers show $1.5 * IQR$ (inter-quartile range), dots indicate outliers.

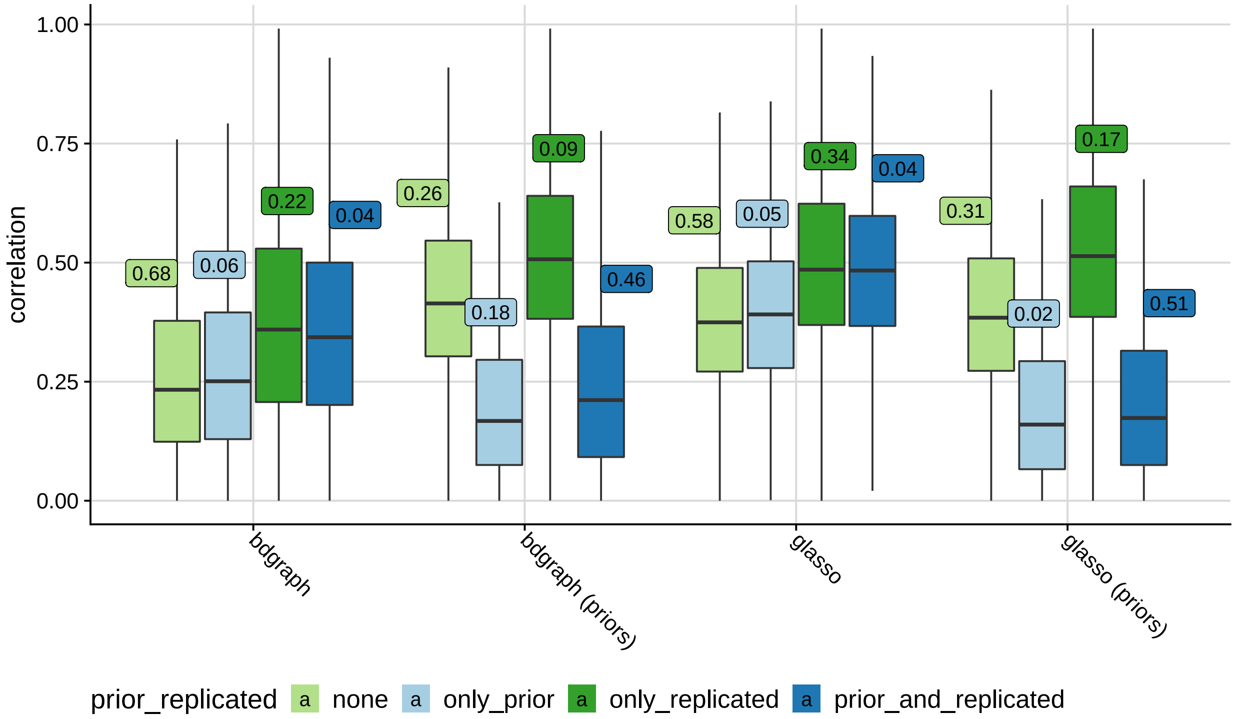

Figure S 10: Analysis of the replication performance of BDgraph and glasso. Shown are results obtained over all gathered loci. X-axis shows different inference methods, y-axis shows distribution of correlation values obtained from the respective second replication cohort; green color indicates presence of prior information and blue color indicates absence of prior information. Dark and light shades of colors indicate replication and no replication of edges, respectively. The numbers annotated to the boxplots indicate the fraction of predicted edges over all inferred networks per graph model which fall into the respective category. Box-plots show medians (horizontal line) and first and third quartiles (lower/upper box borders). Whiskers show $1.5 * IQR$ (inter-quartile range).

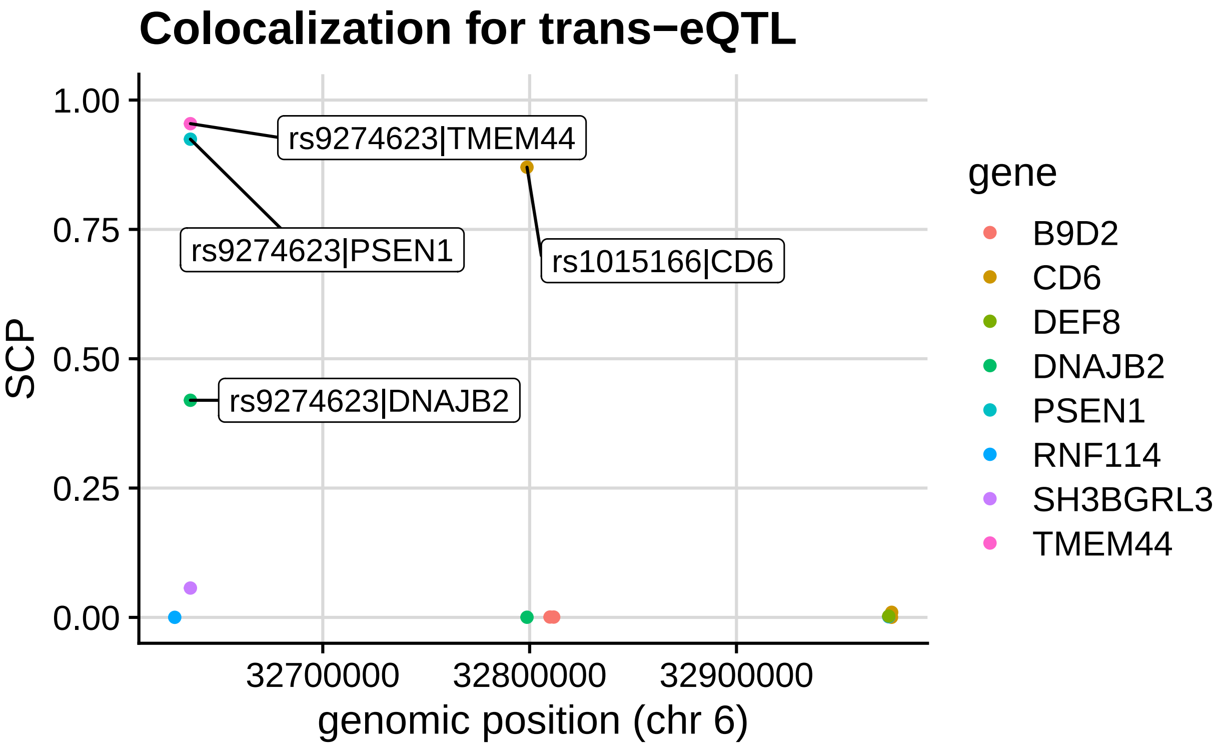

Figure S 11: Results for the colocalization enrichment analysis for the schizophrenia locus. Y-axis shows the SNP-level colocalization probability for individual SNP-Gene pairs, x-axis genomic position of the respective SNPs.

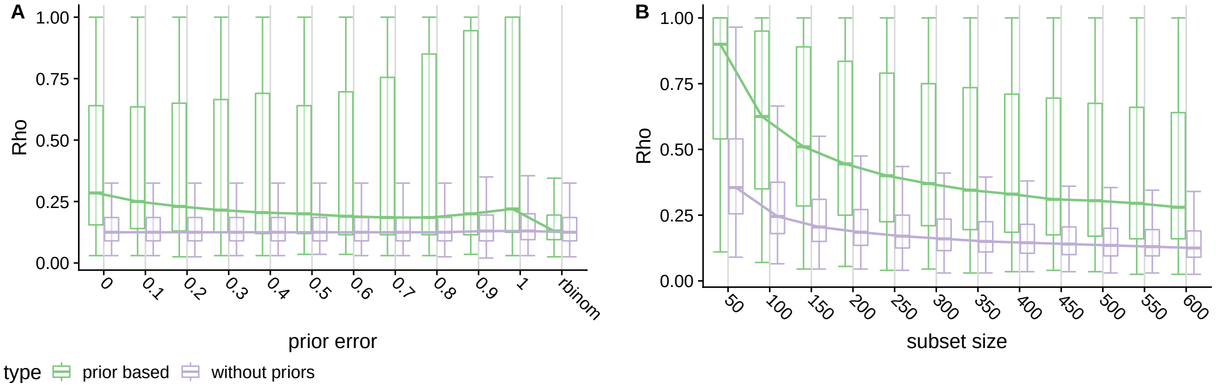

Figure S 12: Rho (glasso penalty parameter) progression when increasing the degree of error in prior information in the simulation study. Shown are results for prior based inference (green) vs prior-agnostic inference (purple). Y-axis shows selected Rho value, x-axis shows increasing degrees of error in prior (A) and increasing subset sizes (B) from the left to the right in the simulated data.

Supplementary Tables

	sentinel	chr	cis_gene	gene_start	gene_end	gene_strand	gene_biotype
1	rs10870226	chr10	TTC40	134621896	134756327	-	protein_coding
2	rs10870226	chr10	RP13-137A17.4	134757471	134778793	-	lincRNA
3	rs10870226	chr10	RP13-137A17.5	134774844	134775741	-	lincRNA
4	rs10870226	chr10	RP13-137A17.6	134779038	134789858	+	lincRNA
5	rs17420384	chr2	AC105393.1	388412	416885	+	lincRNA
6	rs17420384	chr2	AC105393.2	421057	422303	+	lincRNA
7	rs17420384	chr2	AC093326.1	490944	492655	-	lincRNA
8	rs17420384	chr2	AC093326.2	545805	546667	+	lincRNA
9	rs17420384	chr2	AC093326.3	558204	578145	+	lincRNA
10	rs2295981	chr13	LINC00354	112554299	112555490	+	lincRNA
11	rs2295981	chr13	AL136302.1	112563079	112563148	-	miRNA
12	rs2685252	chr2	AC105393.1	388412	416885	+	lincRNA
13	rs2685252	chr2	AC105393.2	421057	422303	+	lincRNA
14	rs2685252	chr2	AC093326.1	490944	492655	-	lincRNA
15	rs2685252	chr2	AC093326.2	545805	546667	+	lincRNA
16	rs2685252	chr2	AC093326.3	558204	578145	+	lincRNA
17	rs57743634	chr5	SDHAP3	1568637	1594735	-	pseudogene
18	rs57743634	chr5	CTD-2012J19.3	1594741	1611582	+	lincRNA
19	rs57743634	chr5	CTD-2012J19.2	1598242	1598362	+	pseudogene
20	rs57743634	chr5	RP11-43F13.1	1599035	1634120	-	pseudogene
21	rs57743634	chr5	CTD-2012J19.1	1614951	1616449	+	pseudogene
22	rs57743634	chr5	MIR4277	1708900	1708983	-	miRNA
23	rs57743634	chr5	CTD-2587M23.1	1725264	1728287	+	lincRNA

Table S 1: Sentinels and their annotated cis genes removed from analysis due to the genes not being measured on the microarrays. Sentinels rs1570038 and rs7924137 did not have any cis genes annotated.

**name**	**version**	**repository**	**attribute**	**reference**
BDgraph	2.61	CRAN	MCMC	Mohammadi and Wit (2015) [?]
gLASSO	1.11	CRAN	Graphical lasso	Friedman *et al.* (2008) [?]
*GENIE*3	1.2.1	bioconductor	Random forests	Huynh-Thu *et al.* (2010) [?]
GeneNet	1.2.13	CRAN	Shrinkage/ FDR	Opgen-Rhein *et al.* (2007) [?]
iRafNet *	1.1-2	CRAN	Random forests	Petralia *et al.* (2015) [?]

Table S 2: Overview of the network inference packages used in the simulation study.
** adjusted to make use of parallel processing, see Methods*

**method**	**R=0**	**R=0.1**	**R=0.2**	**R=0.3**	**R=0.4**	**R=0.5**	**R=0.6**	**R=0.7**	**R=0.8**	**R=0.9**	**R=1**	**R=rbinom**
**bdgraph (priors)**	**0.93**	**0.91**	**0.87**	0.83	0.80	0.77	0.72	0.69	0.64	0.60	0.55	**0.83**
**glasso (priors)**	0.87	0.81	0.74	0.66	0.60	0.53	0.46	0.41	0.34	0.27	0.21	0.42
**bdgraph (empty)**	0.84	0.84	0.84	**0.84**	**0.84**	**0.84**	**0.84**	**0.85**	**0.85**	**0.85**	**0.85**	**0.83**
**bdgraph (full)**	0.84	0.84	0.84	**0.84**	**0.84**	**0.84**	**0.84**	**0.85**	**0.84**	**0.84**	**0.85**	**0.83**
**genenet**	0.65	0.66	0.65	0.65	0.65	0.66	0.66	0.67	0.67	0.67	0.67	0.65
**irafnet**	0.45	0.43	0.42	0.39	0.37	0.36	0.34	0.31	0.28	0.24	0.20	0.42
**glasso**	0.43	0.43	0.43	0.43	0.43	0.43	0.44	0.44	0.44	0.45	0.46	0.42
**genie3**	0.38	0.37	0.37	0.37	0.37	0.38	0.38	0.39	0.39	0.38	0.40	0.35

Table S 3: Table showing an overview over the performance (mean MCC) in the simulation study for each method for all prior error scenarios, sorted by first column. Highest mean MCC for each scenario is indicated in bold.

**method**	**R=0**	**R=0.1**	**R=0.2**	**R=0.3**	**R=0.4**	**R=0.5**	**R=0.6**	**R=0.7**	**R=0.8**	**R=0.9**	**R=1**	**R=rbinom**
**bdgraph (priors)**	**0.93**	**0.91**	**0.87**	**0.84**	0.81	0.77	0.73	0.70	0.65	0.61	0.56	0.82
**glasso (priors)**	0.87	0.81	0.74	0.66	0.60	0.54	0.47	0.42	0.35	0.29	0.22	0.42
**bdgraph (empty)**	0.84	0.84	0.84	**0.84**	**0.84**	**0.84**	**0.84**	**0.84**	**0.84**	**0.84**	**0.84**	**0.83**
**bdgraph (full)**	0.84	0.84	0.84	**0.84**	**0.84**	**0.84**	**0.84**	**0.84**	**0.84**	**0.84**	**0.84**	**0.83**
**genenet**	0.63	0.64	0.63	0.63	0.63	0.64	0.64	0.65	0.65	0.65	0.65	0.63
**glasso**	0.43	0.43	0.43	0.43	0.43	0.43	0.43	0.44	0.44	0.44	0.45	0.42
**irafnet**	0.37	0.35	0.34	0.31	0.29	0.28	0.26	0.23	0.21	0.18	0.16	0.40
**genie3**	0.31	0.30	0.30	0.30	0.30	0.31	0.31	0.32	0.32	0.31	0.31	0.28

Table S 4: Same as ST **??**, but showing mean F1 scores instead of MCC. Highest mean F1 for each scenario is indicated in bold.

	method	R=0	R=0.1	R=0.2	R=0.3	R=0.4	R=0.5	R=0.6	R=0.7	R=0.8	R=0.9	R=1	R=rbinom
1	bdgraph (priors)	1.00	1.00	1.00	0.99	0.99	0.99	0.99	0.99	0.99	0.99	0.98	1.00
2	irafnet	1.00	1.00	1.00	1.00	1.00	1.00	1.00	1.00	1.00	1.00	1.00	0.99
3	bdgraph (empty)	1.00	1.00	1.00	1.00	1.00	1.00	1.00	1.00	1.00	1.00	1.00	1.00
4	bdgraph (full)	1.00	1.00	1.00	1.00	1.00	1.00	1.00	1.00	1.00	1.00	1.00	1.00
5	glasso (priors)	1.00	0.99	0.99	0.98	0.98	0.97	0.97	0.96	0.96	0.96	0.96	0.97
6	genenet	0.99	0.99	0.99	0.99	1.00	0.99	1.00	1.00	1.00	1.00	0.99	0.99
7	glasso	0.98	0.98	0.98	0.98	0.98	0.98	0.98	0.98	0.98	0.98	0.99	0.97
8	genie3	0.96	0.96	0.95	0.95	0.94	0.94	0.94	0.94	0.94	0.94	0.95	0.94

Table S 5: Same as ST **??**, but showing mean specificity scores instead of MCC.

method	N= 50	100	150	200	250	300	350	400	450	500	550	600
**bdgraph (priors)**	**0.86**	**0.86**	**0.87**	**0.89**	**0.90**	**0.91**	**0.92**	**0.92**	**0.92**	**0.93**	**0.93**	**0.93**
**glasso (priors)**	0.83	0.85	0.86	0.86	0.87	0.87	0.87	0.87	0.88	0.88	0.88	0.88
**bdgraph (empty)**	0.43	0.57	0.64	0.69	0.74	0.76	0.78	0.80	0.81	0.82	0.83	0.84
**bdgraph (full)**	0.42	0.56	0.64	0.69	0.74	0.76	0.78	0.80	0.81	0.82	0.83	0.84
**irafnet**	0.32	0.38	0.41	0.42	0.43	0.44	0.44	0.44	0.45	0.45	0.45	0.45
**genenet**	0.29	0.43	0.50	0.54	0.57	0.60	0.61	0.63	0.63	0.65	0.65	0.66
**genie3**	0.26	0.30	0.32	0.33	0.34	0.34	0.35	0.35	0.36	0.36	0.36	0.36
**glasso**	0.20	0.27	0.31	0.34	0.37	0.38	0.39	0.40	0.41	0.42	0.42	0.43

Table S 6: Table showing an overview over the performance (mean MCC) in the simulation study for each method for different sub samplings of simulated data (increasing from left to right), sorted by first column. Highest mean MCC for each scenario is indicated in bold.

method	N= 50	100	150	200	250	300	350	400	450	500	550	600
**bdgraph (priors)**	**0.86**	**0.85**	**0.87**	**0.89**	**0.90**	**0.91**	**0.91**	**0.92**	**0.92**	**0.93**	**0.93**	**0.93**
**glasso (priors)**	0.83	**0.85**	0.86	0.86	0.87	0.87	0.87	0.87	0.88	0.88	0.88	0.88
**bdgraph (empty)**	0.42	0.55	0.63	0.68	0.73	0.75	0.78	0.79	0.81	0.82	0.83	0.84
**bdgraph (full)**	0.41	0.55	0.63	0.68	0.73	0.75	0.78	0.79	0.81	0.82	0.83	0.84
**genenet**	0.24	0.39	0.46	0.51	0.55	0.57	0.59	0.60	0.61	0.63	0.63	0.64
**irafnet**	0.24	0.30	0.32	0.34	0.34	0.35	0.36	0.36	0.36	0.37	0.37	0.37
**glasso**	0.19	0.27	0.31	0.34	0.36	0.38	0.39	0.40	0.41	0.42	0.42	0.43
**genie3**	0.19	0.23	0.24	0.26	0.26	0.27	0.28	0.28	0.28	0.29	0.29	0.29

Table S 7: Table showing an overview over the performance (mean F1) in the simulation study for each method for different sub samplings of simulated data (increasing from left to right), sorted by first column. Highest mean F1 for each scenario is indicated in bold.

	method	N= 50	100	150	200	250	300	350	400	450	500	550	600
1	irafnet	1.00	1.00	1.00	1.00	1.00	1.00	1.00	1.00	1.00	1.00	1.00	1.00
2	bdgraph (priors)	1.00	1.00	1.00	1.00	1.00	1.00	1.00	1.00	1.00	1.00	1.00	1.00
3	glasso (priors)	1.00	1.00	1.00	1.00	1.00	1.00	1.00	1.00	1.00	1.00	1.00	1.00
4	genenet	0.99	0.99	0.99	0.99	1.00	1.00	1.00	1.00	1.00	1.00	1.00	1.00
5	bdgraph (empty)	0.99	0.99	1.00	1.00	1.00	1.00	1.00	1.00	1.00	1.00	1.00	1.00
6	glasso	0.98	0.98	0.98	0.98	0.98	0.98	0.98	0.98	0.97	0.97	0.97	0.97
7	bdgraph (full)	0.97	0.99	0.99	1.00	1.00	1.00	1.00	1.00	1.00	1.00	1.00	1.00
8	genie3	0.95	0.96	0.96	0.96	0.96	0.96	0.96	0.96	0.96	0.96	0.96	0.96

Table S 8: Table showing an overview over the performance (mean specificity) in the simulation study for each method for different sub samplings of simulated data (increasing from left to right), sorted by first column. Highest mean specificity for each scenario is indicated in bold.

TP	TN	FP	FN	F1-score	specificity	sensitivity	MCC	truth	comparison
763	5817721	10936	10233	0.067	0.998	0.069	0	huri	biogrid
660	5174583	8180	8830	0.072	0.998	0.070	0	huri	bioplex_hct
1012	8349931	13990	13438	0.069	0.998	0.070	0	huri	bioplex_hek
763	5817721	10233	10936	0.067	0.998	0.065	0	biogrid	huri
3066	13256192	21445	24358	0.118	0.998	0.112	0	biogrid	bioplex_hct
4231	19838437	33804	34733	0.110	0.998	0.109	0	biogrid	bioplex_hek
660	5174583	8830	8180	0.072	0.998	0.075	0	bioplex_hct	huri
3066	13256192	24358	21445	0.118	0.998	0.125	0	bioplex_hct	biogrid
15629	24136440	32533	25759	0.349	0.999	0.378	0	bioplex_hct	bioplex_hek
1012	8349931	13438	13990	0.069	0.998	0.067	0	bioplex_hek	huri
4231	19838437	34733	33804	0.110	0.998	0.111	0	bioplex_hek	biogrid
15629	24136440	25759	32533	0.349	0.999	0.325	0	bioplex_hek	bioplex_hct

Table S 9: Comparison of reference networks in human including the human reference interactome (HuRI), the network used in this study (biogrid) and two versions of bioplex for the HEK293T (bioplex_hct) and HCT116 (bioplex_hek) cell lines. 'truth' column indicates the set ground truth network for the comparison and 'comparison' the network being compared against the respective set ground truth. TP: true positives; TN: true negatives; FP: false positives; FN: false negatives; MCC: Matthews Correlation Coefficient

	method	Expression	TF activities
1	glasso (priors)	0.74 (0.18)	**0.75 (0.18)**
2	bdgraph (priors)	0.46 (0.1)	**0.49 (0.1)**
3	irafnet	0.36 (0.23)	**0.43 (0.21)**
4	genenet	0.31 (0.12)	**0.33 (0.12)**
5	bdgraph (empty)	0.29 (0.11)	**0.3 (0.12)**
6	glasso	0.25 (0.21)	**0.28 (0.22)**
7	genie3	**0.2 (0.23)**	**0.2 (0.19)**

Table S 10: Table shows the mean cross cohort replication MCC for expression and TF activity based analyses for each method and standard deviations in parentheses. Highest mean MCC per method is indicated in bold.

locus	nodes	#edges	edges (OV)	#ChIP	ChIP (OV)	#PPI	PPI (OV)	#GG	GG (OV)	MCC
rs9859077	95 (89)	399 (287)	137	155	115 (84%)	7	0 (0%)	106	7 (5%)	0.52
rs7783715	25 (17)	28 (23)	6	14	3 (50%)	0	0 (0%)	4	0 (0%)	0.8
rs730775	55 (49)	90 (67)	43	40	40 (93%)	1	0 (0%)	20	1 (2%)	0.66

Table S 11: Comparison of the networks inferred in this study to the networks extracted from [?]. Numbers in brackets indicate statistics for the networks from the original publication or the percent of edges of a specific type (ChIP, PPI, GG) which have been replicated. '#ChIP', '#PPI' and '#GG' columns indicate the total number of edges of the designated type in the inferred network from the current study. ChIP: edges with ChIP-seq evidence; PPI: Edges with protein-protein interaction evidence; GG: gene-gene edges inferred without PPI evidence. '(OV)' columns indicate overlap for respective edge category.
